# Perovskite Solar Cells: A Porous Graphitic Carbon based Hole Transporter/Counter Electrode Material Extracted from an Invasive Plant Species *Eichhornia Crassipes*

**DOI:** 10.1038/s41598-020-62900-4

**Published:** 2020-04-22

**Authors:** Selvakumar Pitchaiya, Nandhakumar Eswaramoorthy, Muthukumarasamy Natarajan, Agilan Santhanam, Vijayshankar Asokan, Venkatraman Madurai Ramakrishnan, Balasundaraprabhu Rangasamy, Senthilarasu Sundaram, Punniamoorthy Ravirajan, Dhayalan Velauthapillai

**Affiliations:** 1 Department of Physics, Coimbatore Institute of Technology, Coimbatore, Tamil Nadu 641 014 India; 2 School of Mechanical Engineering, Vellore Institute of Technology, Vellore, Tamil Nadu 632 014 India; 3 Environmental Inorganic Chemistry, Department of Chemistry and Chemical Engineering, Chalmers University of Technology, 412 96 Göteborg, Sweden; 4 Department of Physics, PSG College of Technology, Coimbatore, Tamil Nadu India; 5 Environment and Sustainability Institute, University of Exeter, Penryn, Cornwall, TR10 9FE United Kingdom; 6 Department of Physics, University of Jaffna, Jaffna, 40000 Sri Lanka; 7 Faculty of Engineering and Science, Western Norway University of Applied Sciences, 5063 Bergen, Norway

**Keywords:** Materials science, Nanoscience and technology, Optical properties and devices, Synthesis of graphene, Devices for energy harvesting, Solar cells

## Abstract

Perovskite solar cells (PSCs) composed of organic polymer-based hole-transporting materials (HTMs) are considered to be an important strategy in improving the device performance, to compete with conventional solar cells. Yet the use of such expensive and unstable HTMs, together with hygroscopic perovskite structure remains a concern – an arguable aspect for the prospect of onsite photovoltaic (PV) application. Herein, we have demonstrated the sustainable fabrication of efficient and air-stable PSCs composed of an invasive plant (*Eichhornia crassipes*) extracted porous graphitic carbon (EC-GC) which plays a dual role as HTM/counter electrode. The changes in annealing temperature (~450 °C, ~850 °C and ~1000 °C) while extracting the EC-GC, made a significant impact on the degree of graphitization - a remarkable criterion in determining the device performance. Hence, the fabricated champion device-1^c^: Glass/FTO/c-TiO_2_/mp-TiO_2_/CH_3_NH_3_PbI_3−x_Cl_x_/EC-GC10@CH_3_NH_3_PbI_3−x_ Cl_x_/EC-GC10) exhibited a PCE of 8.52%. Surprisingly, the introduced EC-GC10 encapsulated perovskite interfacial layer at the perovskite/HTM interface helps in overcoming the moisture degradation of the hygroscopic perovskite layer in which the same champion device-1^c^ evinced better air stability retaining its efficiency ~94.40% for 1000 hours. We believe that this present work on invasive plant extracted carbon playing a dual role, together as an interfacial layer may pave the way towards a reliable perovskite photovoltaic device at low-cost.

## Introduction

Lower cost, shorter payback time and an unprecedented rise in power conversion efficiency (PCE) escalating from 3.8% in 2009 to 24.2% (2019) have turned the attention of researchers and industrial community towards perovskite solar cells (PSCs) within a decade^1–5^. Outstanding photovoltaic properties such as high charge carrier mobility, long electron-hole diffusion length, high absorption coefficient with tuneable bandgap property, low-exciton binding energy, and easy solution preparation techniques make it to compete with traditional commercial silicon solar cells^6,7^. In general, a typical PSC is composed of an electron transport layer (ETL), an active absorbing layer, a HTL, and a counter electrode. Nevertheless, the use of expensive and unstable conducting polymers based hole transporting materials (HTMs) such as (2,2′,7,7′-tetrakis(N,N-di-p-methoxyphenylamine)-9,9′-spirobifluorene (spiro-MeOTAD) and poly [bis(4-phenyl)(2,4,6-trimethylphenyl)amine] (PTAA) which costs around ~$ 500 and ~$ 3,000 per gram respectively and also their instability issues towards thermal and moisture strictly restricts their usage in practical applications^8–13^. Similarly, the use of expensive noble metal electrode precursors such as gold (Au), silver (Ag) and aluminium (Al) with energy-intensive techniques for the deposition of back contact further hinders the onsite commercial production^14–16^.

As to overcome such practical issues, replacing the unstable HTMs and the expensive back contacts with a low-cost alternative material would result in bringing down the overall cost of the PSC devices and also improve their performance^17,18^. Hence, recent researchers found that this instability issue within the PSC devices could be tackled readily by the use of inorganic HTMs, yet the cell performance with an inorganic HTM layer is typically substandard to the one with an organic HTM^17^. This paved the way to introduce a promising low-cost and earth-abundant carbon as an effective replacer for the unstable HTMs and expensive electrodes, as it can play the dual role as hole transporter and as an electrode^19–22^. Also, the carbon materials have an optimal work function value of about −5.0 eV to −5.1 eV which is equal to that of the gold electrode which makes it a promising replacement for achieving improved photovoltaic performance and better air and moisture stability^23,24^. With this, recent researchers have focused to fabricate PSCs using different forms of carbonaceous materials such as graphene, reduced-graphene oxide (rGO), graphdiyne and carbon having different structures of carbons such as nanotubes (SWCNTs & MWCNTs), fullerenes, C_60_, carbon nano-horns, nano-onions, nano-tori, and nano-wall^25–28^. However, the use of carbon based HTMs in PSC fails to achieve superior charge transportation. Hence, it is an important aspect to facilitate charge extraction property which can be achieved by introducing suitable device architecture and by making necessary interface engineering which could play a crucial role in further enhancement of the device stability and efficiency of carbon based PSCs^5,29–31^. After several efforts taken by the recent active researchers, carbon based perovskite device performance has been lifted to the succeeding level by achieving a device efficiency to a PCE of ~21.01%^25^. Yet, they could achieve excellent stability retained up to 70–90% over ~720–800 hrs^24,32^.

With all the above previous reports, the present study focuses to enhance the air and moisture stability of the carbon based PSCs at low-cost for the onsite photovoltaic application. Herein, we have demonstrated the facile preparation of graphitic carbon from an invasive waste plant (*Eichhornia Crassipes*) which can be effectively used as an efficient hole transporter/back electrode – a dual role in PSCs. In general, *Eichhornia Crassipes* (Family: Pontederiaceae) commonly called water hyacinth is an aquatic weed that can easily spread and interfere in an ecosystem by posing a serious threat to the native biodiversity, leading to economic loss worldwide. Moreover, the influence of this plant on freshwater results in a waning of oxygen level which leads to the death of huge aquatic plants and animals. Furthermore, it also results in a drop in temperature, pH value, and nutrient levels. In fact, this *Eichhornia Crassipes* plant has been included by the world conservation union (IUCN) in the list of 100 most dangerous invasive species^33^.

To our knowledge, this is the first report on the preparation of a low-cost porous graphitic carbon extracted from an invasive plant species for the sustainable fabrication of hole transport material/counter electrode for efficient enhancement of air and moisture stability in PSCs. Also, the graphitization nature of the prepared carbon has been found to be improved for the three different annealing temperatures (~450 °C, ~850 °C and ~1000 °C). Correspondingly, a graphitic carbon encapsulated perovskite (EC-GC10@CH_3_NH_3_PbI_3−x_Cl_x_) interfacial layer has been introduced between the perovskite/carbon-HTM junction (prepared by mixing the extracted graphitic carbon (I_D_/I_G_ = 0.75) having a high surface area of 1000.452 m^2^/g into the perovskite solution). Finally, the three different annealed porous graphitic carbon HTM/electrode has been deposited using a facile brush painting technique for the fabrication of PSC device with structure: FTO/c-TiO_2_/mp-TiO_2_/CH_3_NH_3_PbI_3−x_Cl_x_/EC-GC10@CH_3_NH_3_PbI_3−x_Cl_x_/EC-GC4/8/10.

## Results and Discussion

### Structural analysis of the plant extracted porous graphitic carbon

#### XRD Analysis

**Figure 1:**
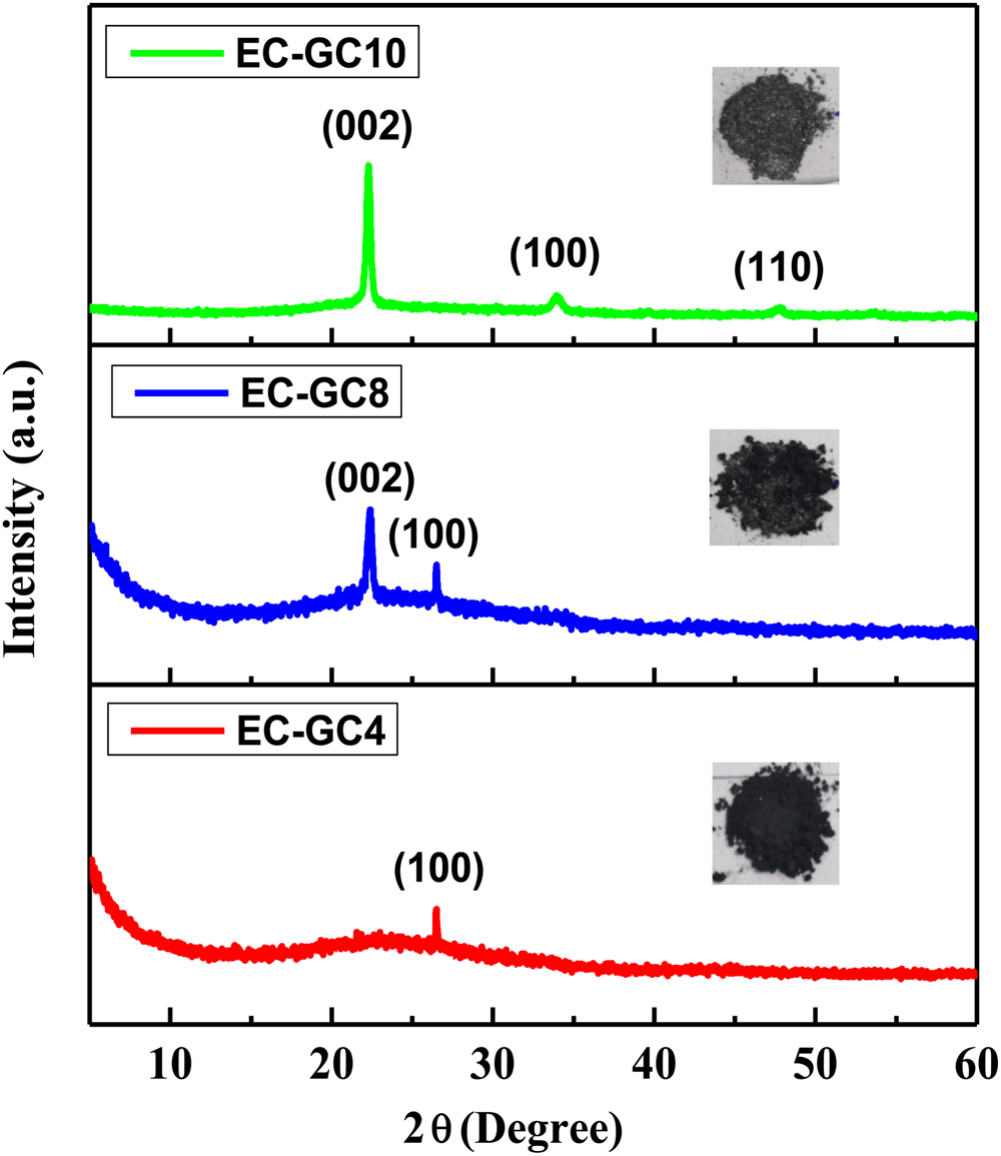
Structural analysis of the porous graphitic carbon materials extracted from an invasive plant species of *Eichhornia Crassipes* using different annealing temperatures ~450 °C (EC-GC4), ~850 °C (EC-GC8) and ~1000 °C (EC-GC10).

The structural properties of the graphitic carbon extracted from an invasive plant waste by a simple green approach technique have been studied using the X-Ray diffraction (XRD) method. The XRD pattern for the prepared samples EC-GC4, EC-GC8 and EC-GC10 are shown in Fig. 1. In this, the diffraction peak at 2θ = 26.50° for the ~450 °C annealed sample (EC-GC4) corresponds to the (100) plane. When the annealing temperature increases to ~850 °C (EC-GC8), a new peak is observed at 2θ value (22.27°) which corresponds to graphitic hexagonal (002) plane^34,35^. The additional new peak confirms that the graphitization process has occurred in the sample EC-GC8. Further, the sample EC-GC10 annealed at the temperature of ~1000 °C, displays three dominant diffraction peaks at 2θ = 22.27°, 33.91° and 47.67° which corresponds to the reflections from (002), (100) and (110) planes respectively. In this, the intensity of the graphitic plane (002) is more when compared to that of the EC-GC8 sample and a shift in the 2θ value from 26.50° to 33.91° of the (100) plane is observed, signifying the improved crystallization of the graphitic phase^36,37^. The increase in the peak intensity reveals that the samples EC-GC8 and EC-GC10 annealed at ~850 °C and ~1000 °C achieved a better crystalline nature than the sample EC-GC4 prepared at ~450 °C. The (002) peak indicates the presence of graphitic carbon,  and the (002) peak comes from the sp^2^-hybridized carbon. With an increase in annealing temperature from ~850 °C to ~1000 °C, the (002) peak becomes more prominent, indicating the improvement of the graphitization degree with respect to the annealing temperature. This graphitic crystalline nature might have been enhanced due to the removal of impurities and ash from the sample because of annealing effect^38^. Further, Fig. S1 depicts the FT-IR spectra for the porous graphitic carbon materials EC-GC4, EC-GC8 and EC-GC10 synthesized using different annealing temperatures.

#### Raman analysis

**Figure 2:**
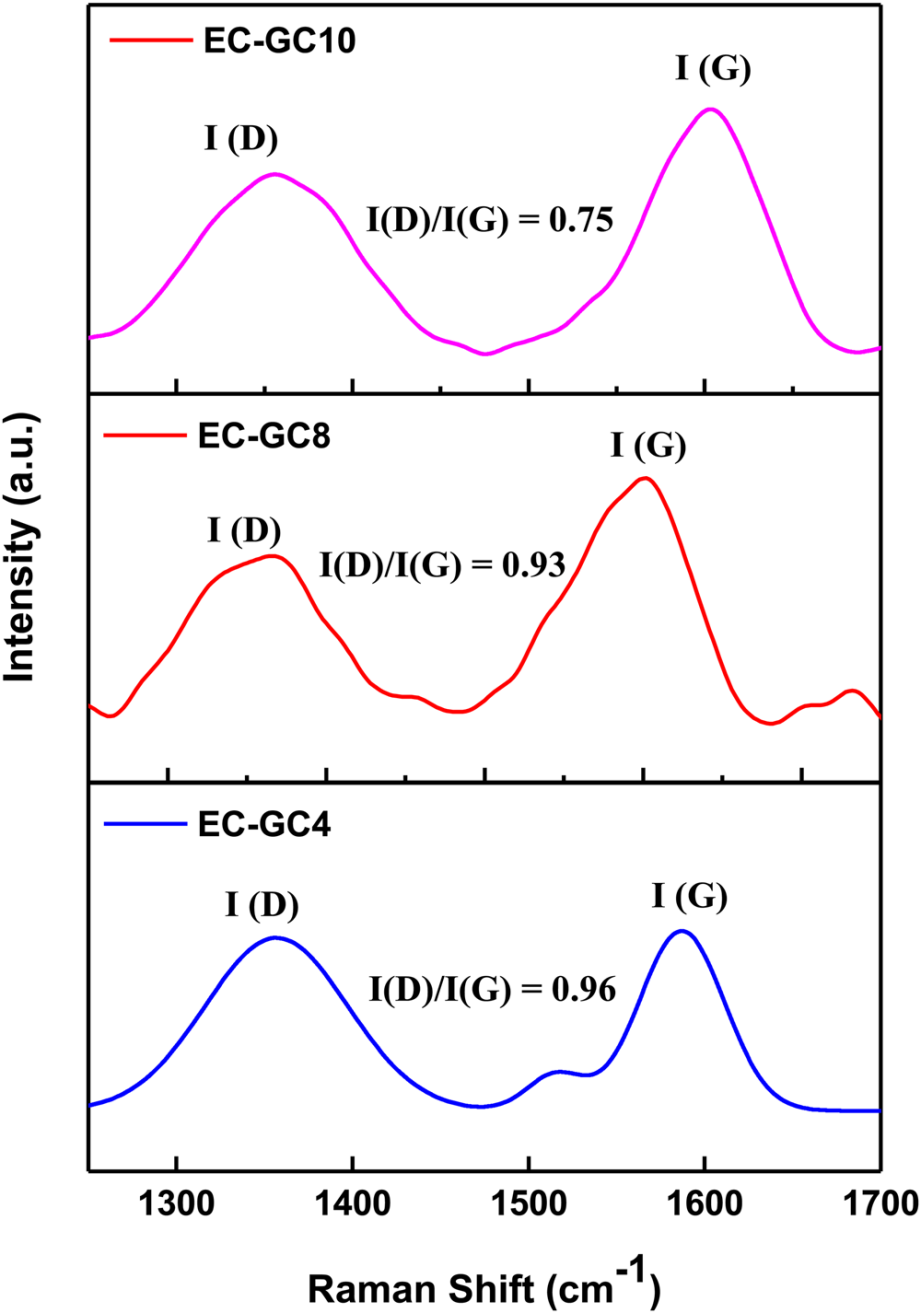
Raman spectra of the porous graphitic carbon materials (**a**) EC-GC4, (**b**) EC-GC8 and (**c**) EC-GC10 synthesized using different annealing temperatures.

Raman spectroscopy is found to be an effective and accurate identification method than XRD in identifying the amorphous and crystalline nature of the several types of carbon such as diamond, graphite, diamond-like carbon, and carbon nitride, etc., and to ascertain the amount of defects in carbon materials due to the structural changes in the graphitic microstructures^39,40^. The peak position and the heights of the prepared graphitic carbon (EC-GC4, EC-GC8, and EC-GC10) samples analyzed using Raman spectra shown in Fig. 2, comprises of two predominant bands namely D-band (D for the disorder) and G-band (G for Graphite). Indeed, the G peak centered at 1586 cm^−1^, 1600 cm^−1^ and 1603 cm^−1^ for samples EC-GC4, EC-GC8 and EC-GC10 respectively correspond to the vibration of sp^2^ hybridized carbon atoms which indicates the existence of nanocrystalline graphitic carbon atoms^41^. Conversely, the peak found at 1354 cm^−1^, 1358 cm^−1^ and 1356 cm^−1^ for the samples EC-GC4, EC-GC8 and EC-GC10 respectively attribute to the defect induced disordered graphitic (D-band) microstructures corresponding to the vibrations of sp^3^ and sp carbon atoms^42^. Consequently, the ratio of D to G peak intensities (I_D_/I_G_) was measured to probe the defects in the prepared graphitic carbon samples. The I_D_/I_G_ value of the prepared samples was found to be 0.96, 0.93 and 0.75 respectively. Hence, the observed changes in the height and sharpness of the D and G peak with respect to the annealing temperature reveals the graphitic nature of the prepared samples. As the I_D_/I_G_ value of the EC-GC10 is found to be less when compared to that of the other samples, it suggests that ~1000 °C annealed sample has sp^2^ hybridization with a high degree of graphitization. The as-prepared porous graphitic carbon indicates the existence of nanocrystalline graphite, which could provide more active sites which in turn facilitates the charge transfer behavior in carbon microstructures. The obtained Raman spectra correlates with the XRD results of the prepared EC-GC samples and the observed structural property with good crystallinity may be usefully used in the PSCs for improving the device performance^43^.

#### FT-IR spectroscopic analysis

Fourier Transform Infra-Red (FT-IR) spectroscopy has been used to detect the functional groups present in the bio-extracted porous graphitic carbon samples EC-GC4, EC-GC8 and EC-GC10 annealed at different temperatures (~450 °C, ~850 °C, ~1000 °C). Several FT-IR predominant bands present in all the samples at 3706 cm^−1^, 1558 cm^−1^, 1229 cm^−1^, 1035 cm^−1^ are assigned to the stretching vibrations of OH, C–H, symmetrical & asymmetrical C=O, and =C–O–C groups respectively and is shown in Fig. S1.

#### Conductivity measurements

Conductivity measurements for the prepared graphitic carbon samples have been carried out under room temperature using a Hall Effect measurement (ECOPIA, HMS 2000) system. Annealing graphitic carbon samples increases the fraction of sp^2^ hybridized carbon which influences the nature of charge transport. An increase in conductivity with respect to the annealing temperature is witnessed in Table 1, which also confirms that these are influenced by the thermal and structural ordering. XRD and Raman results confirm the increasing order of graphitization with respect to the annealing temperature^44,45^. From the observed conductivity tests, the positive sign of bulk concentration indicates that the majority carriers are holes and it is a p-type material. The mobility of charge carriers is found to increase with increase in annealing temperature. The resistivity value is observed to decrease with increase in annealing temperature and the values are 1.49 × 10^−2^ Ω cm, 1.46 × 10^−2^ Ω cm, 8.68 × 10^−3^ Ω cm as shown in Fig. S2. For the higher annealing temperature in the range of 1000 °C, the material has attained higher conductivity and it can be used in the fabrication of PSCs for achieving higher efficiency.

**Table 1:** Conductivity measurement results of the porous graphitic carbon samples annealed at different temperatures.

Annealing Temperature	Sample Name	Bulk Concentration (N_b_) (cm^−3^)	Sheet Concentration (N_s_) (cm^−2^)	Mobility (µ) (cm^2^V^−1^s^−1^)	Resistivity (ρ) (Ω cm)	Conductivity (${\boldsymbol{\sigma }}$) (Siemens/cm)
450 °C	EC-GC4	2.37 × 10^16^	2.36 × 10^14^	20.37	1.49 × 10^−2^	67.33
850 °C	EC-GC8	6.06 × 10^19^	1.20 × 10^16^	37.81	1.46 × 10^−2^	68.05
1000 °C	EC-GC10	1.9 × 10^21^	9.21 × 10^15^	76.95	8.68 × 10^−3^	115.20

### Morphological and compositional analysis of the porous graphitic carbon

**Figure 3:**
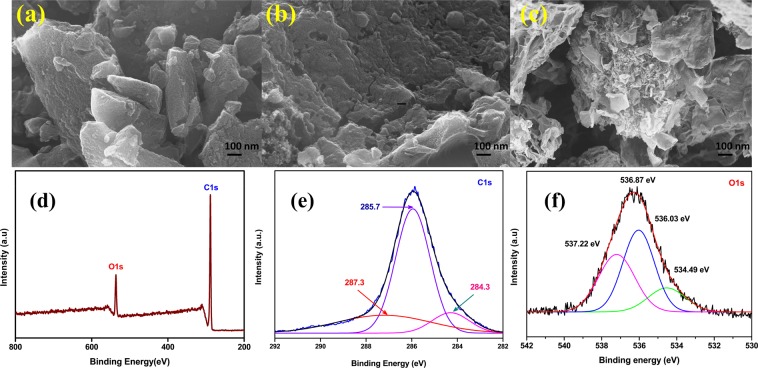
Morphological and compositional analysis of the porous graphitic carbon. (**a–c**) FE-SEM images of porous graphitic carbon material synthesized at different temperatures of ~450 °C (EC-GC4), ~850 °C (EC-GC8) and ~1000 °C (EC-GC10) respectively. XPS analysis of EC-GC10 porous graphitic carbon powder (**d**) Survey spectrum (**e**,**f**) high-resolution C1s and O1s core level spectrum.

The morphology of the bio-prepared porous graphitic carbon annealed at different temperatures (~450 °C, ~850 °C and ~1000 °C) has been investigated using FESEM analysis. Figure 3(a) shows the formation of an aggregated and bulky graphitic carbon for the ~450 °C annealed sample and this may be due to the presence of unreacted residuals of the *Eichhornia crassipes* plant bio-char^8^. The increase in the graphitization temperature to ~850 °C, shows the formation of a fibrous structure with improved porosity and a decrease in the particle size of the graphitic carbon which is due to the evaporation of moisture content in the bio-char and this is shown in Fig. 3(b). When the annealing temperature increases to ~1000 °C, the fibrous structured samples apparently modify into a thin layer of flake like structured graphitic carbon as shown in Fig. 3(c). This is expected, because the purpose of using higher annealing temperature is not only for the removal of the unwanted impurities but also to improve the porosity in the EC-GC materials, and this is also observed in the nitrogen sorption analysis. The obtained morphology of EC-GC10 may be helpful in rapid charge transport and could improve the stability and PCE in PSCs.

X-ray photoelectron spectroscopy (XPS) analysis was carried out to study the elemental composition and to analyze the chemical states of the elements present in the prepared EC-GC10 sample. Figure 3(d), shows the survey spectrum (200–800 eV) of the graphitic carbon sample, which has peaks corresponding to C1_S_ (288.9 eV) and O1_S_ (536.87 eV). Figure 3(e), displays the C1_S_ spectrum recorded in the range from 282 to 292 eV and has been deconvoluted and shown as three dominant peaks. The presence of peaks centered at 284.3 eV, 285.7 eV and 287.3 eV corresponds to sp^2^ C=C, sp^3^ C–C and C=O respectively, and this reveals the presence of hybridized graphitic carbon^41,46^. The presence of a dominant oxygen peak centered at 536.87 eV is shown in Fig. 3(f).

### Brunauer-Emmett-Teller (BET) analysis of the porous graphitic carbon

**Figure 4:**
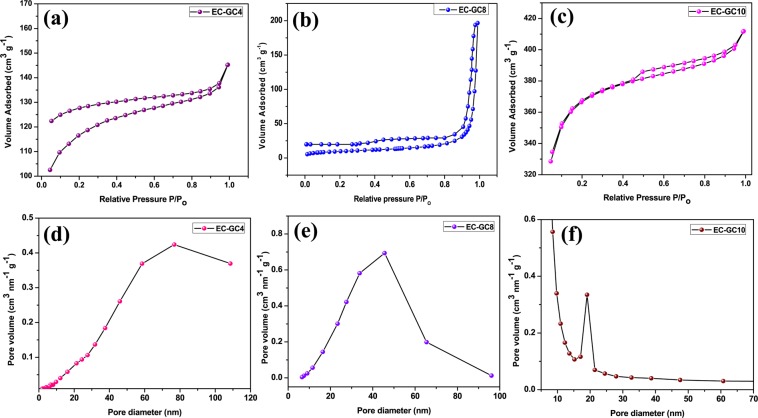
N_2_ adsorption/desorption isotherm plots of (**a**) EC-GC4 (**b**) EC-GC8 (**c**) EC-GC10 using BET analysis and (**d–f**) shows the respective pore size distribution.

The specific surface area and the corresponding porosity of the bio-prepared graphitic carbon were investigated using N_2_ adsorption/desorption isotherms and BJH analysis and are shown in Fig. 4(a–f). The relevant parameters extracted from the isotherms are given in Table S2, along with a comparison with some previous reports on the surface area, pore size and volume of graphitic carbon extracted from various bio-waste materials. The sample (EC-GC4) annealed at ~450 °C shows a type IV isotherm with the hysteresis loop of H2^47,48^. The observed specific surface area (Fig. 4(a)) was found to be 23.94 m^2^g^−1^. From the BJH analysis shown in Fig. 4(d), the average pore size and volume have been calculated and are found to be 77.3 nm and 0.29 cm^3^g^−1^ respectively and the results observed shows the presence of microporous nature^49^. Whereas the isotherm of EC-GC8 shown in Fig. 4(b) is a characteristic of a typical mesoporous-like material, which is confirmed by the type IV plateau with an H3 hysteresis loop^41,50,51^. In this, we could observe a higher surface area of 329.99 m^2^g^−1^ and the material is mesoporous with pore size and volume of 45.8 nm and 0.11 cm^3^g^−1^ respectively **(**Fig. 4(e)). The BET analysis of the porous graphitic carbon sample annealed at ~1000 °C (EC-GC10) shows type II isotherm in H4 loop with enhanced surface area (1000.45 m^2^g^−1^) and mesoporous nature with the presence of narrow slit-like pores (pore size of 19.9 nm and pore volume of 0.23 cm^3^g^−1^ **(**Fig. 4(c,f))^52^. The BJH pore size distribution with mesoporous nature and high specific surface area of the sample EC-GC10 demonstrates that the thermal reduction with loss of oxygen functional group has created more porosity when compared to that of the other two samples and this may be due to high-temperature annealing^53^. The above-obtained BET reports suggest that the sample having a high specific surface area with mesoporous nature may play a significant role and can also afford ideal pathways that will improve current density in PSC device^54^.

### Optical, the structural and morphological analysis of the prepared CH_3_NH_3_PbI_3−x_Cl_x_

**Figure 5:**
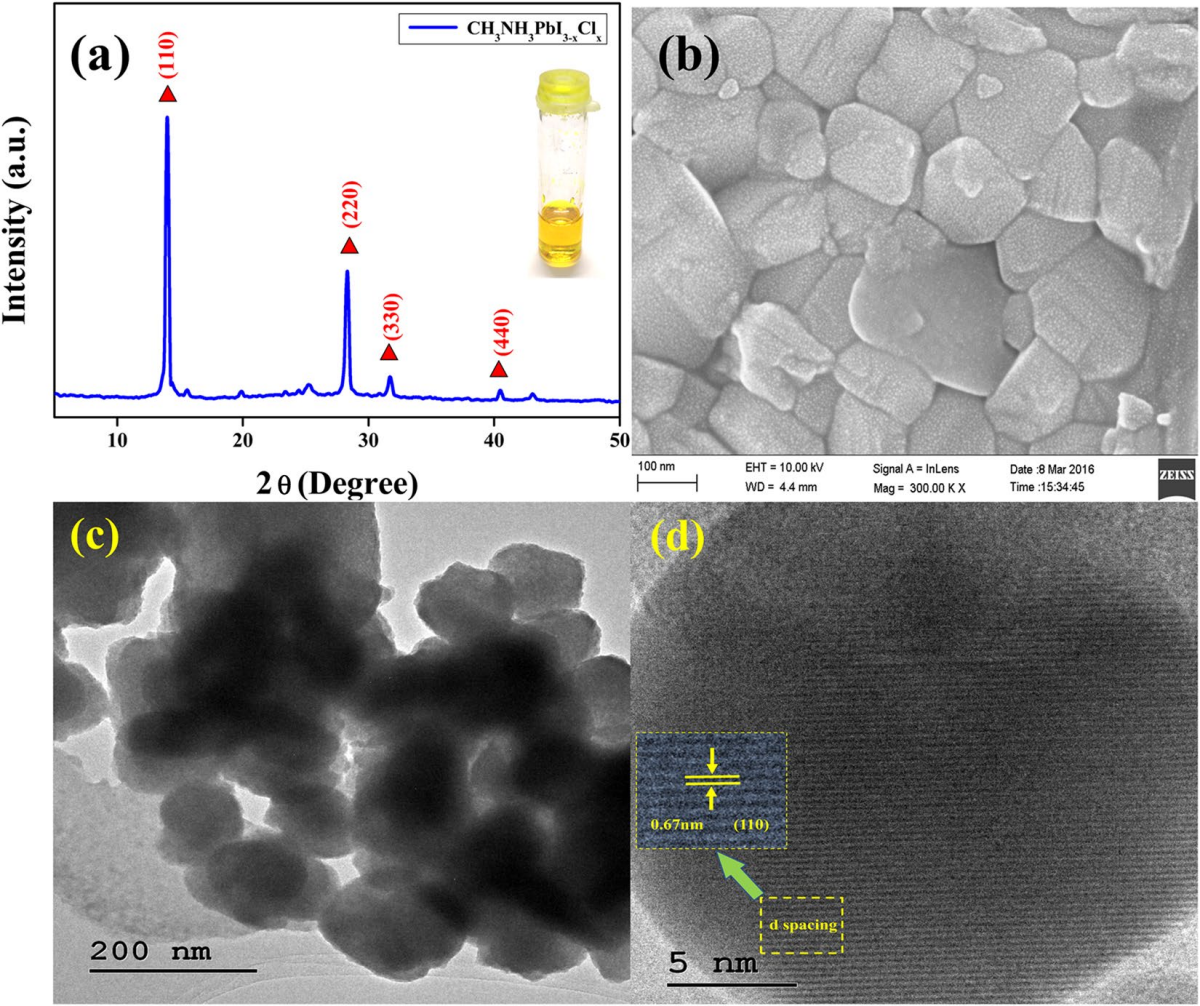
Structural and morphological analysis of CH_3_NH_3_PbI_3−x_Cl_x_ perovskite. (**a**,**b**) XRD pattern and FE-SEM image of mixed halide perovskite sample (CH_3_NH_3_PbI_3−x_Cl_x_) and (inset a) shows the photograph of the homogenous perovskite precursor solution (**c**) high resolution microscopic TEM image of CH_3_NH_3_PbI_3−x_Cl_x_ perovskite and (**d**) magnified HRTEM image with lattice fringes.

The structural properties of the prepared CH_3_NH_3_PbI_3−x_Cl_x_ and the EC-GC encapsulated perovskites were analyzed using XRD analysis. The obtained diffraction pattern of CH_3_NH_3_PbI_3−x_Cl_x_ perovskite is shown in Fig. 5(a). The intense peaks centered at 13.97°, 28.35°, 31.76°, and 40.42° correspond to (110), (220), (330) and (440) planes respectively and all the obtained peaks are in accordance with the previously reported works on methylammonium lead halide perovskites.

Some previous reports on the addition of excess chlorine ions into the perovskite structure have mentioned that this has resulted in providing a significant impact on their morphology with improved crystallization which in turn plays a pivot role in achieving better photovoltaic performance^55–57^. The present work proposes a plausible inclusion of stoichiometric ‘Cl’ ions using a CH_3_NH_3_Cl precursor into the CH_3_NH_3_PbI_3−x_Cl_x_ perovskites. The optical properties of the prepared mixed halide perovskite (CH_3_NH_3_PbI_3−x_Cl_x_) has been depicted in the Supplementary Information Figs. S3 and S4. The surface morphology of the prepared perovskite after the addition of CH_3_NH_3_Cl has been depicted in Fig. 5(b). Furthermore, high-resolution transmission electron microscopic (HRTEM) images have also been used to analyze the morphological features and are depicted in Fig. 5(c,d). The results show, the presence of a well-crystallized polygonal perovskite structure having a ‘d’ spacing value of about 0.67 nm, corresponding to the (110) plane which correlates with the plane value observed in XRD result. Moreover, the results observed in the present work also correlate with the previous works of Qi *et al*. and Spencer *et al*., in which they have stated that the incorporation of chlorine specifically has an impact on the structural and morphological properties of the perovskite films, which could result in achieving an improved optoelectronic characteristics and this may help in achieving an enhanced PV performance^58,59^.

### Structural and morphological analysis of the prepared EC-GC10 encapsulated CH_3_NH_3_PbI_3−x_Cl_x_

The formation mechanism of EC-GC encapsulated perovskite is shown in Fig. S5(a,b). Though the mixed halide perovskite resulted in achieving improved device efficiency, its poor stability and degradation phenomena under the ambient environment have been a tailback for the commercial development of long-term stability PSCs^60–62^. As to control the degradation of perovskite structure due to moisture, heat, light and ion migrations^63^, several remedial measures have been reported which can make the perovskite structure to withstand under ambient conditions^64^ and some of them are like incorporation and post-treatment of perovskite material using inorganic compounds^65^, composition engineering using mixed anions and cations^66^ and also by introducing carbon-based materials^60,64^. Hence in the present study, an attempt has been made to overcome the above-mentioned drawbacks by encapsulating the perovskite material using carbon-based materials which could be used as an interfacial layer sandwiched between the active absorber and as prepared graphitic carbon counter electrode.

**Figure 6:**
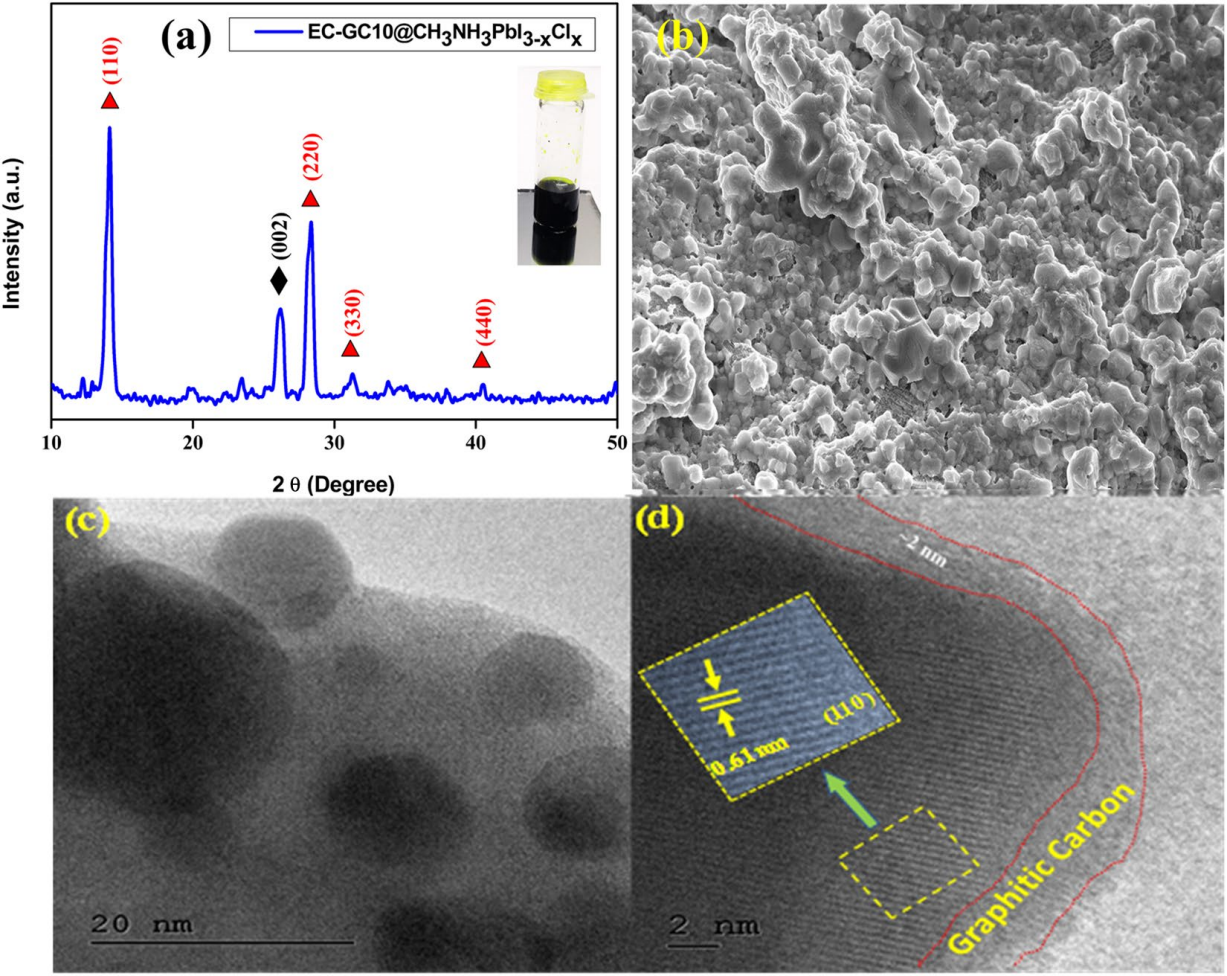
Structural and morphological analysis of EC-GC10 encapsulated CH_3_NH_3_PbI_3−x_Cl_x_ perovskite material. (**a**,**b**) XRD pattern and FE-SEM image of graphitic carbon material encapsulated perovskite (inset a) shows the photograph of the heterogeneous EC-GC10@CH_3_NH_3_PbI_3−x_Cl_x_ precursor solution (**c**) high resolution microscopic TEM image of EC-GC10 (~2 nm) encapsulated perovskite materials and (**d**) magnified HR-TEM image with lattice fringes of EC-GC10@CH_3_NH_3_PbI_3−x_Cl_x_.

The diffraction pattern of the graphitic carbon encapsulated perovskite (EC-GC10@CH_3_NH_3_PbI_3−x_Cl_x_) is shown in Fig. 6(a). In this, an additional diffraction peak found at 2θ = 26.12° (002 plane), could be ascribed to the existence of the hexagonal graphitic carbon [JCPDS file no 75–1621]^67^. The diffraction peaks at 2θ = 13.97°, 28.35°, 31.76°, and 40.42° corresponding to (110), (220), (330) and (440) planes respectively of tetragonal perovskite structure were also observed. Here the intensity of all the perovskite peaks is comparatively less when compared to that of pure perovskite peaks, which could be due to the occupation of the graphitic carbon atoms in the apical sites of the perovskite, this also substantiates the formation of encapsulated EC-GC10@CH_3_NH_3_PbI_3−x_Cl_x_ structure. Due to the availability of 6p electron in the Pb^2+^sites, there is strong coordination between Pb^2+^ ion with C=O group in the porous graphitic carbon, and this is believed to be the reason behind the encapsulation mechanism^68,69^ and this  is illustrated in the schematic diagram given in Fig. S6(c). These results are in well agreement with the previous report of Lokesh Saini *et al*., and they have reported about the synthesized graphitic carbon folded Ni cubic metallic phase, annealed at 700 °C temperature in nitrogen atmosphere formed as Ni/Graphitic carbon core-shell structure^70^. Similarly, Yongping Liao *et al*., have also reported about the crystalline graphitic carbon/Fe_3_C nanocomposite used as a counter electrode for solar cell applications^71^. The photograph of the prepared carbon encapsulated perovskite solution has been given in the inset of Fig. 6(a). The observed FESEM morphology of the EC-GC10@CH_3_NH_3_PbI_3−x_Cl_x_ which has been illustrated in Fig. 6(b) shows the formation of a coagulated island structure of perovskite material encapsulated by the EC-GC.

From the high-resolution TEM images shown in Fig. 6(c,d), the perovskite crystals can be clearly seen in the darker region entrapped inside the capsules of 2 nm size lighter contrast graphitic carbon which has been marked with red dotted lines. The magnified images of the highlighted region show the lattice fringes having a ‘d’ spacing value of 0.61 nm, corresponding to the (110) interplanar spacing of tetragonal perovskite crystal plane which correlates well with the XRD results. From this, we could observe that the fringe spacing of perovskite obtained in the EC-GC10@CH_3_NH_3_PbI_3−x_Cl_x_ is less when compared to that of the CH_3_NH_3_PbI_3−x_Cl_x_ perovskite layer (0.67 nm) and this reduction may be due to the encapsulation obtained using graphitic carbon. This accumulative morphological evolution of the EC-GC10@CH_3_NH_3_PbI_3−x_Cl_x_ layer may be attributed to the slow crystallization induced by the encapsulation of porous graphitic carbon.

### Current density – voltage characteristics of perovskite solar cells

Figure S6(a) illustrates the schematic representation of energy level work function diagram of the fabricated PSC device structure and (b) cross-sectional FESEM image of fabricated carbon HTM based PSCs.

**Figure 7:**
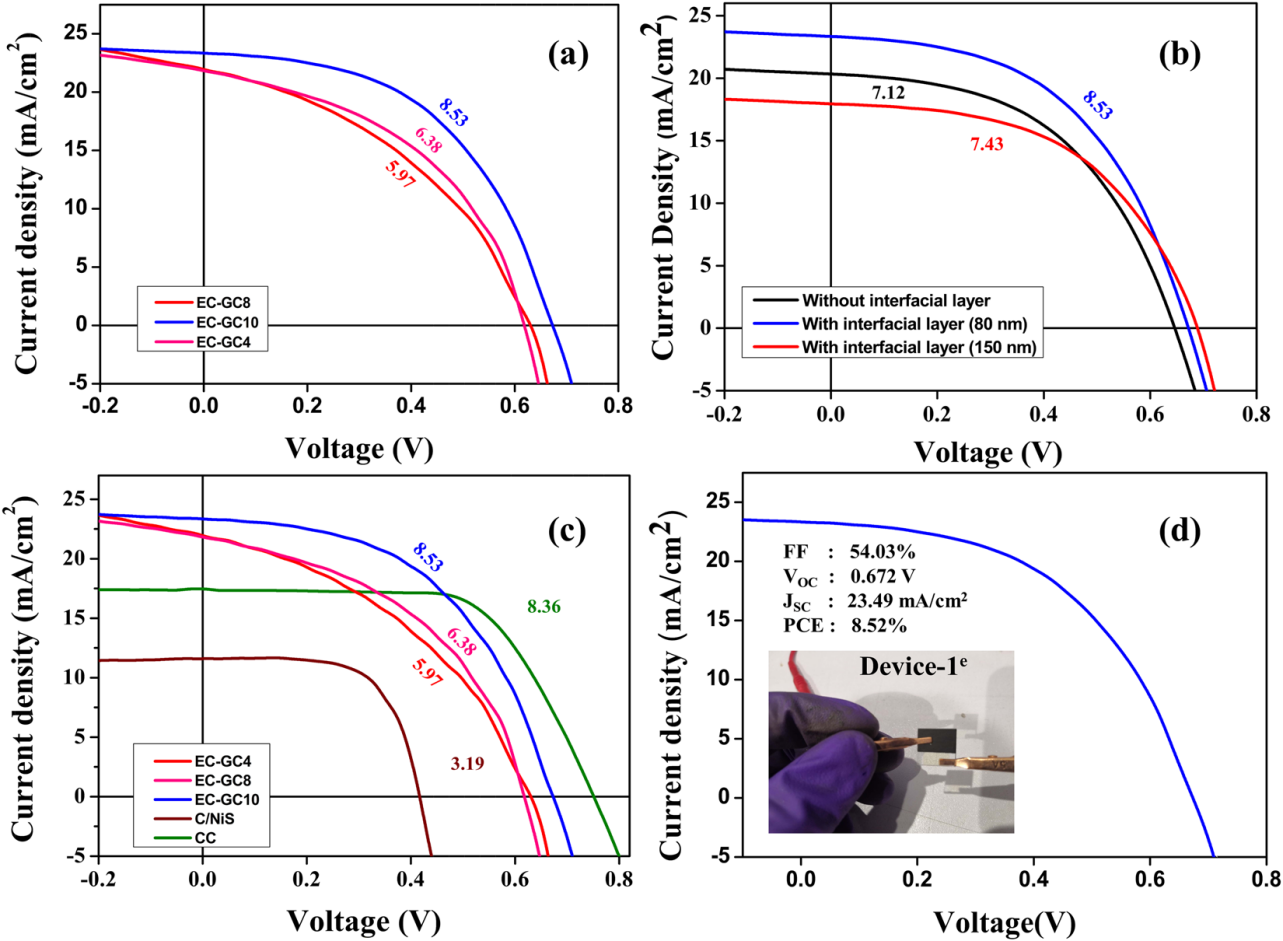
Photovoltaic performance of Carbon HTMs based PSCs. (**a**) Current density-voltage (J–V) characteristics of the invasive plant synthesized graphitic porous carbon annealed at different temperature (EC-GC4, EC-GC8 and EC-GC10) based hole transport material PSC devices. (**b**) J–V curves of devices without and with interfacial layer of 80 nm and 150 nm thickness (EC-GC10@CH_3_NH_3_PbI_3−x_Cl_x_). (**c**) Comparative study of the J–V characteristics  of the different carbon EC-GC4, EC-GC8, EG-GC10, C/NiS and CC based HTM PSC devices. (**d**) J–V characteristics of the champion device-1^c^ and the inset shows the photograph of the fabricated PSC.

Figure 7(a) displays the photocurrent density-voltage (J-V) curves of glass/FTO/c-TiO_2_/mp-TiO_2_/CH_3_NH_3_PbI_3−x_Cl_x_/EC-GC10@CH_3_NH_3_PbI_3−x_Cl_x_/C-HTMs) based perovskite devices fabricated using the EC-GC materials  annealed at different temperatures (EC-GC4, EC-GC8, and EC-GC10). Table 2 summarizes all the corresponding key photovoltaic parameters (J_SC_, V_OC,_ and FF) extracted from the J-V curves, The results demonstrate that the device constructed using EC-GC10 has a high PCE of 8.52% with a short current density (J_SC_) of 23.49 mA/cm^2^, an open-circuit voltage (V_OC_) of 0.672 V and a fill factor (FF) of 54.03%. Whereas the other two devices fabricated using EC-GC4 and EC-GC8 (device-1^a^ and device-1^b^**)** exhibit a PCE of 5.97% and 6.38% respectively. As seen, the V_OC_ of the perovskite devices (0.631 V, 0.620 V, and 0.672 V) constructed using different EC-GC materials almost remains unchanged but there is a change in the current density values. In fact, the role of annealing temperature greatly influences the hexagonal graphitic phases and the porosity of the as-prepared carbon materials and this may be the reason behind the enhancement of J_SC_ (22.01 mA/cm^2^, 21.96 mA/cm^2^, and 23.49 mA/cm^2^) and FF (43.01%, 46.80%, and 54.03%) of the perovskite devices. The better device performance of the EC-GC10 based device can be directly ascribed to the larger surface area (1000.45 m^2^g^−1^) with a pore size of 19.9 nm and a pore volume of 0.23 cm^3^g^−1^ as seen from the BET results.

**Table 2:** Typical Photovoltaic parameters of PSCs fabricated using different carbon based hole extraction layer. Note: SC-Spin Coating, SP-Spray Pyrolysis, BP-Brush Painting.

Device Structure	HTL	V_OC_ (V)	J_SC_ (mA/cm^2^)	FF (%)	PCE (%)	PCE (%) after 1000 hrs.	Deterioration (%) for 1000 hrs.
Device-1^a^	EC-GC4	0.631	22.01	43.01	5.97	3.56	40.38
Device-1^b^	EC-GC8	0.620	21.96	46.80	6.38	4.34	31.88
Device-1^c^	EC-GC10	0.672	23.49	54.03	8.52	8.05	05.57
Device-1^d^	C/NiS	0.417	11.63	65.69	3.19	1.60	49.65
Device-1^e^	CC	0.749	17.51	63.77	8.36	6.73	19.52
Device-1^f^	Without Interfacial Layer	0.649	20.50	53.51	7.12	2.62	63.30
Device-1^g^	Interfacial Layer (150 nm)	0.719	18.81	55.05	7.43	—	—

^a^FTO/c-TiO_2_^(SP)^/mp-TiO_2_^(SC)^/CH_3_NH_3_PbI_3−x_Cl_x_^(SC)^/EC-GC10@CH_3_NH_3_PbI_3−x_Cl_x_^(SC)^/EC-GC4^(BP)^.

^b^FTO/c-TiO_2_^(SP)^/mp-TiO_2_^(SC)^/CH_3_NH_3_PbI_3−x_Cl_x_^(SC)^/EC-GC10@CH_3_NH_3_PbI_3−x_Cl_x_^(SC)^/EC-GC8^(BP)^.

^c^FTO/c-TiO_2_^(SP)^/mp-TiO_2_^(SC)^/CH_3_NH_3_PbI_3−x_Cl_x_^(SC)^/80 nm - EC-GC10@CH_3_NH_3_PbI_3−x_Cl_x_^(SC)^/EC-GC10^(BP)^.

^d^FTO/c-TiO_2_^(SP)^/mp-TiO_2_^(SC)^/CH_3_NH_3_PbI_3−x_Cl_x_^(SC)^/EC-GC4@CH_3_NH_3_PbI_3−x_Cl_x_^(SC)^/C/NiS^(BP)^.

^e^FTO/c-TiO_2_^(SP)^/mp-TiO_2_^(SC)^/CH_3_NH_3_PbI_3−x_Cl_x_^(SC)^/EC-GC4@CH_3_NH_3_PbI_3−x_Cl_x_^(SC)^/CC^(BP)^.

^f^FTO/c-TiO_2_^(SP)^/mp-TiO_2_^(SC)^/CH_3_NH_3_PbI_3−x_Cl_x_^(SC)^/EC-GC10^(BP)^.

^g^FTO/c-TiO_2_^(SP)^/mp-TiO_2_^(SC)^/CH_3_NH_3_PbI_3−x_Cl_x_^(SC)^/150 nm - EC-GC10@CH_3_NH_3_PbI_3−x_Cl_x_^(SC)^/EC-GC10^(BP)^.

Figure 7(b) shows the J-V curves of the perovskite devices fabricated with and without porous graphitic carbon encapsulated CH_3_NH_3_PbI_3−x_Cl_x_ interfacial layer and the respective photovoltaic parameters obtained for the device-1^c^ and device-1^f^ are given in Table S1. The device fabricated without an interfacial layer exhibited a lower PCE of 7.12% with V_OC_ (0.649 V), J_SC_ (20.50 mA/cm^2^) and FF (53.51%) when compared to that of the one with an interfacial layer (device-1^c^). The obtained PCE difference may be attributed to the better charge collection and lower recombination phenomena exhibited by the introduced interfacial layer in the fabricated device structure^72,73^. A similar report by Xiaowen *et al*., shows that a better interfacial contact promotes fluent charge transfer at the interface and leads to a higher FF value for carbon-based PSCs^74^. This reveals that the interfacial engineering layer is essential and has been used in the present work for preventing recombination^75^.

Although the interfacial layer plays a crucial role in determining the PCE with its effective charge collection capabilities, the previous reports have not focussed much on some of the key parameters such as their thickness which could have a significant effect on the device performance^72^. In this, the necessity and the importance of optimizing the thickness of the interfacial layer has been discussed. As to understand the role of the interfacial layer (EC-GC10@CH_3_NH_3_PbI_3−x_Cl_x_) thickness in the device performance, a series of devices have been fabricated in this work. The thickness of the interfacial layer of the devices was changed using different spin rotation speeds. The J-V characteristics of the two devices fabricated using the interfacial layer formed with the thickness of 80 and 150 nm are also shown in Fig. 7(b) and their detailed photovoltaic parameters are summarized in Table 2. From this result analysis, it could be found that the maximal performance was achieved when the thickness of the interfacial layer is at 80 nm. Here the EC-GC10@CH_3_NH_3_PbI_3−x_Cl_x_ interfacial layer is expected to effectively intercalate into the grain boundaries of the deposited perovskite layer. This resulted in enabling both fast charge transfer (to the back electrode) and reduction in diffusion length needed (boosting the short-circuit current). A decline in the J_SC_ value of device with 150 nm thick interfacial layer compared to the 80 nm thick interfacial layer, maybe due to the increase in the diffusion length of the device having the higher interfacial layer thickness, however, the obtained V_OC_ for this device is higher due to the lower surface recombination^76^. On the other hand, for the device without the interfacial layer, the decrease in V_OC_ and FF may be attributed to the larger resistance induced at the perovskite/electrode interfaces^77^. Therefore, the optimized thickness of the EC-GC10@CH_3_NH_3_PbI_3−x_Cl_x_ interfacial layer in the present work was found to be at 80 nm.

In addition to this, Fig. 7(c) shows the comparative J-V curves of the carbon/nickel sulphide composite based HTM devices (device-1^d^) and commercial carbon based device(device-1^e^) along with the prepared EC-GC hole transport based devices. From the J-V curves, it could be inferred that the CC based HTM device shows an enhanced V_OC_ (0.749 V) and FF (63.77%) compared to that of all the other devices and this may be mainly due to the presence of small graphite flakes and carbon black which has better conductivity^24,78,79^ and tends to provide much more favorable interfacial contact sites between the perovskite and the back electrode which improves the charge transport behavior^17^. In general, the used commercial carbon paste contains TiO_2_ nanoparticles as a binder along with graphite and carbon black^80^. With this, the lowering of HOMO energy level (with respect to the vacuum level) of the hole transport material in perovskite solar cells has been found to increase its open circuit voltage (V_OC_)^81^. As the HOMO level of TiO_2_ is lower compared to carbon, here the increase in open circuit voltage for the commercial carbon paste basded device could be due to this shift induced by the addition of TiO_2_. The fabricated devices-1^a–c^ exhibited a higher ‘J_SC_’ which may be mainly due to the increased charge carrier mobility resulting from the reduction in the recombination of electron-hole pairs. Herein, the introduced bio extracted porous graphitic carbon may act as a key factor in facilitating charge extraction/injection effectively at the perovskite/HTM interface and thereby enhances both the efficiency and stability of the fabricated devices^82^. Similarly, the obtained J-V results have been compared with our previously reported work on PSCs fabricated using an inorganic nickel sulphide carbon composite (carbon/NiS) based HTM as they have a favorable deep-lying HOMO (valence band edge value) with work function from 5 eV–5.6 eV^49^. It has been found that the device with highly transparent carbon/NiS used as a counter electrode achieved a higher FF (65.69%) compared to all the other demonstrated devices but exhibited only a low PCE of 3.19%. Further, some of the recent reports based on perovskite solar cells fabricated using different carbon-based HTMs are given in Table S3 and have been compared with the champion device-1^c^ fabricated in the present work.

The PSCs fabricated in the present report have a cell size of 1 cm × 1 cm and is shown in the inset of Fig. 7(d). In it, a black mask with an active area of 0.314 sq.cm has been used for measuring the photovoltaic performance under simulated AM 1.5 solar irradiation at an intensity of 100 mW/cm^2^ measured at room temperature and the champion device-1^c^ of the lot, exhibited a PCE of 8.52% under ambient atmosphere.

### Device stability test under ambient condition

The previous reports on organic conducting polymer-based HTMs in PSCs indicate that it has the potential to compete with the conventional solar cells, yet the device stability remains a big concern due to its vital aspect for the onsite photovoltaic application^83^. Hence, the air and moisture stability tests were also carried out on the prepared porous graphitic carbon-based PSCs (they were exposed directly to light and air under the ambient environment). Overall, the fabricated PSC devices using different carbon-based HTMs were exposed to an ambient environment (at 25° ± 5 °C with 70 ± 5% humidity) and their stability was tested for ~1000 hours.

A comparative study was carried out on the analysis of device stability and deterioration chart for the devices with and without EC-GC10 encapsulated perovskite interfacial layer is depicted in Fig. S7(a,b). It could be inferred from the deterioration test that the device having an interfacial layer (device-1^c^) showed better stability with a deterioration percentage of just 5.60% exhibiting a PCE of 8.05% after ~1000 hours. Whereas the stability of the device fabricated without the interfacial layer (device-1^f^) encountered a deterioration of more than 63.30% displaying a PCE of 2.62% after 1000 hours. This lower deterioration value for the device-1^c^ compared to that of the device-1^f^, could be attributed to the better interfacial contact between EC-GC encapsulated perovskite/EC-GC based HTM which facilitates hole transfer at the interface and prevents the degradation of perovskite material against air and moisture which resulted in achieving long-term stability^72,84,85^. Hence, all the devices fabricated in the present work consists of an EC-GC10 encapsulated perovskite interfacial layer.

**Figure 8:**
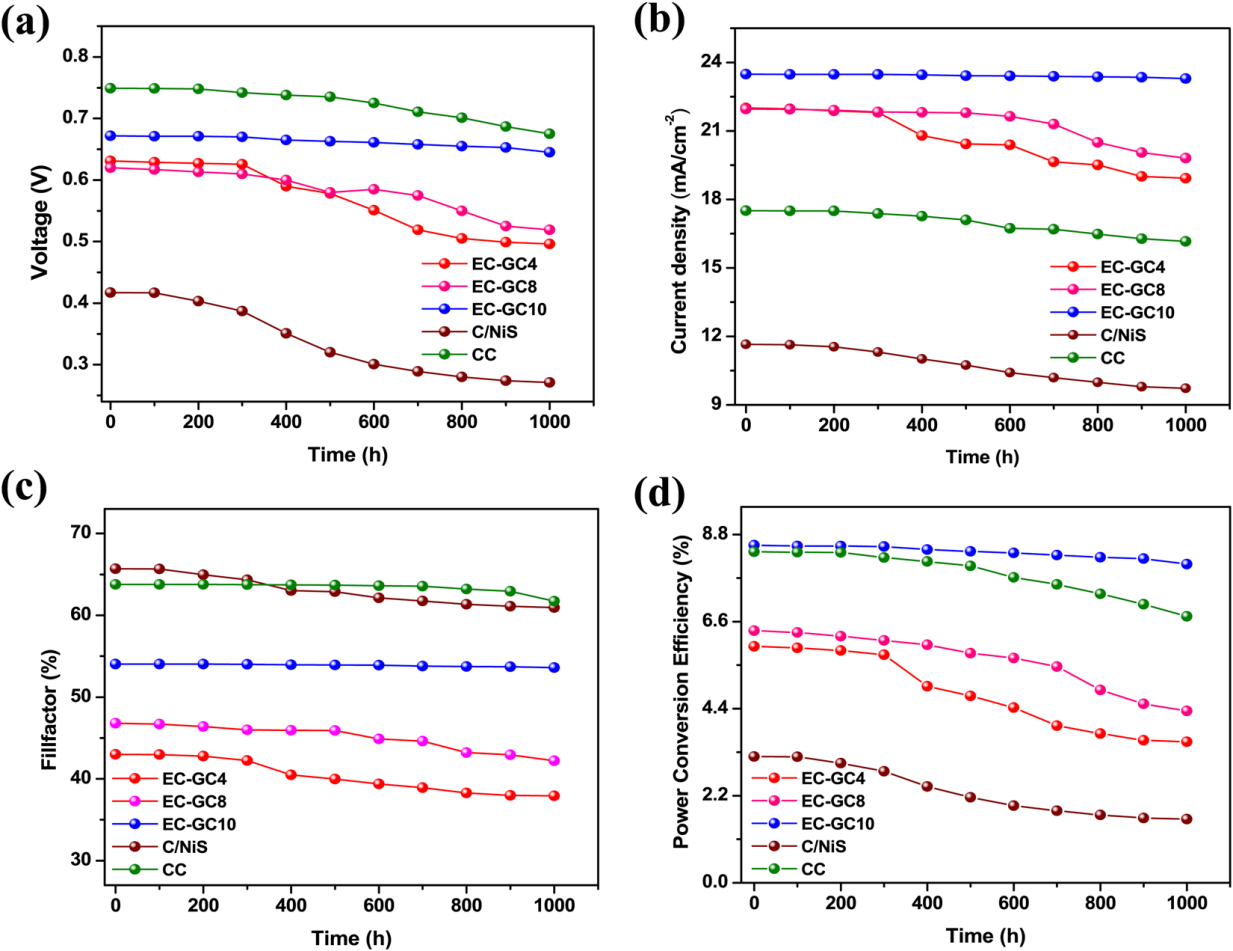
A comparative study of the parameters of device stability in terms of (**a**) V_OC_ (**b**) J_SC_ (**c**) FF and (**d**) PCE for the PSC devices fabricated using different HTMs (EC-GC4, EC-GC8, EG-GC10, C/NiS and CC carbon) under ambient room condition (25 ± 5 °C, 70 ± 5% humidity).

**Figure 9:**
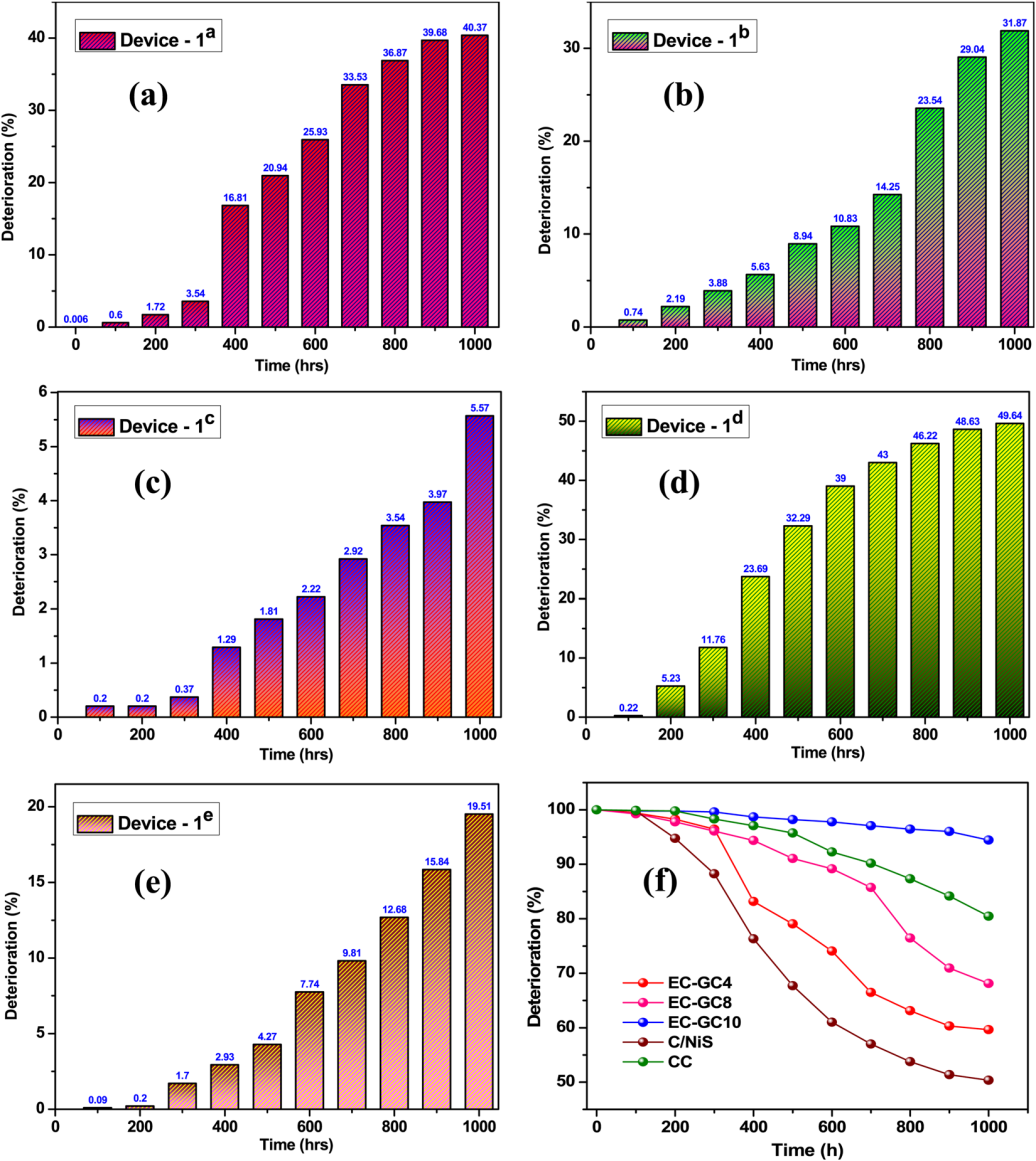
Deterioration chart of different carbon based PSC devices (**a**) EC-GC4 in Device-1^a^ (**b**) EC-GC8 in Device-1^b^ (**c**) EC-GC10 in Device-1^c^ (**d**) C/NiS in Device-1^d^ (**e**) CC in Device-1^e^ (**f**) Comparative stability study of all the PSCs fabricated using different carbon based HTMs (EC-GC4, EC-GC8, EG-GC10, C/NiS and CC carbon) and tested for 1000 hours under ambient room condition (25 ± 5 °C, 70 ± 5% humidity).

The devices fabricated using EC-GC4, EC-GC8 and EC-GC10 have been named as device-1^a^, device-1^b^, and device-1^c^ respectively. As a comparison, we have also fabricated carbon/nickel sulphide (C/NiS) and commercial carbon (CC) HTMs based PSCs which were named as device-1^d^ and device-1^e^ respectively. The plots shown in Fig. 8(a–d), describes the device stability in terms of (a) V_OC_ (b) J_SC_ (c) FF and (d) PCE for PSC devices fabricated using different HTMs. Figure 9(a–f) shows the deterioration chart for different carbon-based PSC Devices (a) EC-GC4 in Device-1^a^ (b) EC-GC8 in Device-1^b^ (c) EC-GC10 in Device-1^c^ (d) C/NiS in Device-1^d^ (e) CC in Device-1^e^ (f) and comparative stability study of all the PSCs fabricated using different carbon-based HTMs (EC-GC4, EC-GC8, EG-GC10, C/NiS, and CC carbon). The deterioration of the devices, in terms of PCE for all the devices, have been calculated using the equation given below,

$${\rm{Deterioration}}\,{\rm{of}}\,{\rm{PCE}}\,{\rm{value}}\,( \% )=\frac{decrease\,in\,PCE\,value}{initial\,PCE\,value\,}\times 100$$

Interestingly all the devices remained stable and showed a minimal deterioration in PCE value just about ~0.3% for 100 hours. Subsequently, all the devices were tested after 500 hours, the PCE value of the device-1^a^ dropped exponentially from ~5.97% to ~4.71% (~20.80% deterioration of PCE) and the device-1^b^ showed a deterioration value of ~8.68% (from PCE of ~6.37% to ~5.80%), whereas the device-1^c^ was almost stable and showed a mere drop in its efficiency from ~8.52% to ~8.37% (only ~0.41% deterioration of PCE). Similarly, the stability of the device-1^e^ was measured for 500 hours and it exhibited a deterioration value of just ~4.10% (PCE of ~8.0%) from its initial original efficiency (~8.36% of PCE) and for the device-1^d^, the deterioration of PCE value was found to be ~32.29% (with PCE of 2.16%). Further, the long-term stability of all the devices was examined by exposing the device for 1000 hours. The PCE value of the device-1^a^ shows a severe decline of up to ~41.38% with PCE of ~3.53%, J_SC_ of ~18.93 mA/cm^2^, V_OC_ of ~0.496 V and FF of ~37.87%. The device-1^b^ displays average long-term stability with reduction in PCE value to ~4.34% having J_SC_ of ~19.81 mA/cm^2^, V_OC_ of ~0.519 V and FF of ~42.22% from its initial efficiency of ~6.37% (~31.88% of reduction in PCE). The device-1^c^ showed substantial and long-term stability up to 1000 hours compared to that of the other devices-1^a,b,d–f^, retaining its deterioration value to less than ~5.57% with a PCE of ~8.05% from its original efficiency (~8.52%). In this case, there is a slight decrease in all the key parameters (J_SC_ ~23.29 mA/cm^2^, V_OC_ ~0.645 V and FF ~53.61%). The results observed in the previous reports state that the degree of graphitization of the graphitic carbon determines the long-term stability for the carbon based HTMs in perovskite solar cells^86^. Similar results were observed in the present work which shows that the prepared EC-GC10 graphitic carbon device showed a high degree of graphitization compared to the other (EC-GC4 < EC-GC8 < EC-GC10) devices and hence exhibited more stability against air and moisture (device-1^a^ < device-1^b^ < device-1^c^). The comparative device-1^e^ constructed using ‘CC’ as HTM, upholds its stability by more than ~80% (degraded from PCE of ~8.35% to ~6.73%) for 1000 hours. Whereas, the stability of the device-1^d^ fabricated using C/NiS composite deteriorated to ~50% after 1000 hrs (~1.60% PCE) from its original PCE of ~3.19% which showed a faster deterioration rate than all the other devices and this may be attributed to the highly unstable behaviour of nickel sulphide to the ambient environment. From the stability tests, it is clear that the bio extracted porous graphitic carbon annealed at 1000 °C (EC-GC10) fabricated PSCs showed better stability performance, upholding the PCE value up to 94% of the initial PCE even after 1000 hrs compared to all the fabricated devices.

## Conclusion

In the present study, cost-effective and environmentally beneficial porous graphitic carbon has been synthesized from an invasive plant species of *Eichhornia crassipes* and has been used for the sustainable fabrication of HTM/electrode in PSCs. The champion device exhibited a maximum PCE of 8.52% which may be attributed to the structural (graphitic nature) and morphological (porous nature) properties of the bio-prepared graphitic carbon. In addition, the introduced EC-GC encapsulated perovskite interfacial layer, helps in fighting the moisture degradation of CH_3_NH_3_PbI_3−x_Cl_x_ - perovskite structure and in reducing the recombination loss at the perovskite/HTM interface. The champion device exhibited better air stability, retaining its PCE up to 94.40%  of its initial PCE value even after 1000 hours, when exposed to the ambient environment and this could be attributed to the degree of graphitization obtained on annealing. The better performance of the fabricated devices against air and moisture is attributed to the naturally extracted EC-GC material which helps in facilitating the charge extraction/injection effectively at the perovskite/HTM junction. As a summary, an invasive plant extracted carbon has been used to play a dual role as HTM and as an electrode, and also together as an interfacial layer in a perovskite device.

## Materials and Methods

### Materials

Methylamine solution (33 wt. % in ethanol), hydroiodic acid (57 wt. % in H_2_O), lead (II) chloride (98%), ethanol (ACS reagent, 99.9%), diethyl ether and N, N-Dimethylformamide (DMF), Titanium di isopropoxide bis (acetylacetonate) (75 wt. % in isopropanol), Dyesol 18 NR Titania paste, fluorine-doped tin oxide plates (FTO) with surface resistivity ~10 Ω/sq, commercial carbon paste (solaronix), α-terpineol, and chlorobenzene (99.8%) were purchased from Sigma-Aldrich and used as received. Invasive plant *Eichhornia crassipes* were collected from the Coimbatore region, Tamilnadu, India for the preparation of porous graphitic carbon.

### Synthesis of bio extracted porous graphitic carbon materials

Bio extraction of porous graphitic carbon (EC-GC) material from an invasive plant species of *Eichhornia crassipes* is a two-step process; (i) preparation of biochar and (ii) extraction of porous graphitic carbon from the prepared biochar.

### Preparation of biochar from the invasive plant

The biochar from the invasive plant was prepared using the ‘ancient Indian oil lamp heating method’. In this, the stems and leaves of the *Eichhornia crassipes* (EC) plants were taken as the precursors for the preparation of biochar. Then the ECs plant parts were chopped into tiny pieces and were washed with deionized water and ethanol at 60 ± 2 °C to remove all the unwanted impurities. Then the cleaned ECs were dried under shadow for 2 days and then dried in direct sunlight for 12 hours and further, the ECs pieces were heated overnight using the ancient Indian oil lamp method. In this low-temperature carbonization process, the invasive ECs have been converted into black colored biochar which could be used further for the extraction of porous graphitic carbon materials.

### Extraction of graphitic carbon from the biochar material

Herein the obtained black colored biochar is graphitized by a two-step process that involves an acid treatment and is followed by a heat treatment at different temperatures in the N_2_ atmosphere. To start with, 3M of the hydrochloric acid solution was added to the above-mentioned biochar and was kept undisturbed for 6 hours at room temperature and the solution was washed several times with DD water to remove unreacted biochar and to maintain pH level at ~7. Further, the biochar was dried at 90 ± 3 °C for overnight. The obtained black colored powder was mixed well-using agate mortar and then annealed at ~450 °C, ~850 °C and ~1000 °C temperature for 3 hours in an argon atmosphere and the final obtained porous graphitic carbon samples were labeled as EC-GC4, EC-GC8, and EC-GC10 respectively.

### Preparation of interfacial layer solution

For the preparation of mixed halide perovskite solution, methylammonium iodide (CH_3_NH_3_I) was synthesized according to our previously reported work^87^. From this, 349.7 mg of CH_3_NH_3_I was taken and dissolved in 1 ml of dimethylformamide (DMF). Then 0.203 mg of lead (II) chloride (PbCl_2_) was slowly added and the mixture was stirred and heated at 90 ± 2 °C for 2 hours and the obtained final mixed halide perovskite (CH_3_NH_3_PbI_3−x_Cl_x_) solution was filtered through a 0.2 μm syringe filter. Further, the CH_3_NH_3_PbI_3−x_Cl_x_ solution was stirred vigorously with 0.001 g of EC-GC10 porous graphitic carbon sample dissolved in chlorobenzene and was ultrasonicated for 10 minutes. The obtained final heterogeneous mixture solution was kept for further process to be used as an interfacial layer of PSCs.

### Perovskite solar cell fabrication

Commercially available fluorine-doped tin oxide (FTO) conductive glass substrates (surface resistivity of 10 Ω/square) were cleaned by ultrasonication in acetone, methanol and then in isopropanol sequentially for 10 minutes at 80 ± 2 °C and finally dried using nitrogen airflow. An electron transporting compact (c-TiO_2_) layer solution was prepared by mixing 700 μL of titanium di isopropoxide bis(acetylacetonate) in 7.0 mL of absolute ethanol (1:10 volume ratio) and was filtered through 0.2 μm pore syringe filter. This solution (1200 µl of above solution for 6 samples) was sprayed on the preheated glass/FTO surface followed by annealing at 450 ± 5 °C for 10 minutes. It was then allowed to cool down to room temperature (RT) and sonicated in absolute ethanol for 20 minutes. The purchased dyesol 18 NR TiO_2_ paste mixed with absolute ethanol (1:3.5 volume ratio) solution and was spin-coated at 4000 rpm for 20 s to form a mesoporous TiO_2_ (mp-TiO_2_) on the top of the c-TiO_2_ layer. It was then annealed at 450 ± 5 °C for 30 minutes. The overnight heated perovskite solution at 70 ± 2 °C was taken and spin-coated on the top of the pre-heated (80 ± 2 °C) glass/FTO/c-TiO_2_/mp-TiO_2_ film at 2000 rpm for the 30 s. The deposited perovskite film was heated at 120 ± 3 °C for 45 minutes so as to remove the organic solvents and this resulted in a systematic color change in the layer from yellow to black confirming the formation of perovskite active layer. A heterogenous graphitic carbon (EC-GC10) mixed perovskite solution was taken and spin-coated at 3500/1500 rpm for 20 s and was heated at 80 ± 2 °C, and this resulted in the formation of porous graphitic carbon encapsulated perovskite (EC-GC10@CH_3_NH_3_PbI_3−x_Cl_x_) thin film with the thickness of 80/150 nm respectively. Finally, the carbon-based hole transporting material (C-HTMs) such as EC-GC4, EC-GC8, EC-GC10, commercial carbon paste (CC) and carbon nickel sulphide composite (C/NiS) was mixed with α-terpineol and chlorobenzene (1:2 vol.%) and coated via brush painting technique on the top of the perovskite layer. The PSC device structure fabricated in the present work are of the form Glass/FTO/c-TiO_2_/mp-TiO_2_/CH_3_NH_3_PbI_3−x_Cl_x_/EC-GC10@CH_3_NH_3_PbI_3−x_Cl_x_/EC-GC based HTMs prepared at different annealed temperatures.

### Characterization techniques

The crystalline structure of the prepared samples was analyzed using an X-ray diffractometer (Bruker D8 ADVANCE ECO). Fourier transform infrared (FTIR) spectrum in the range of 4000–500 cm^−1^ has been recorded using the Bruker FTIR spectrometer. Jasco V-670 was used to record the UV-Visible absorption spectra. Perkin Elmer LS 45 was used to record the photoluminescence (PL) studies. The morphology and the microstructure of the samples were recorded using Zeiss, Sigma, UK (Field-emission scanning electron microscope) and JEOL JEM 2100 (High-resolution transmission electron microscope) instruments. ESCA2000 of VG micro-tech, UK was used to record X-ray photoelectron spectroscopy (XPS) spectra . BET analysis was carried out using ASAP 2020 micromeritics. Renishaw, UK, Model: Invia was used to perform micro Raman spectroscopy studies. The current density-voltage (J-V) measurements were carried out using Keithley 2420 source meter under 1 Sun illumination with AM 1.5 G filter from Newport, ORIEL Solar Simulator^88^. The J-V scan was made from V_OC_ −1.5 to +1.5 V with a time delay at 30 ms with a step size of 10 mV and a scan rate of 50 mV/s^87^. At least three sets were taken for observing photovoltaic behavior such as current density-voltage (J-V) and the device stability for 1000 hours at 25° ± 5 °C at 70 ± 5% humidity. A black mask of 0.314 sq.cm as an active area was used on top of the cell to avoid the overestimation of current density when excess light falls on the device outside the active area.

## Supplementary information

Supplementary Information.
