# Supplementary material for: Perovskite Solar Cells: A Porous Graphitic Carbon based Hole Transporter/Counter Electrode Material Extracted from an Invasive Plant Species Eichhornia Crassipes
Source: Sci Rep. 2020 Apr 22;10:6835. doi: 10.1038/s41598-020-62900-4 (PMC7176691; doi:10.1038/s41598-020-62900-4)
Supplement: Supplementary file 1 — Supplementary Information. [file 41598_2020_62900_MOESM1_ESM.docx]

**Supporting Information**

**Perovskite Solar Cells: A Porous Graphitic Carbon based Hole Transporter/Counter Electrode Material Extracted from an Invasive Plant Species *Eichhornia Crassipes***

Pitchaiya Selvakumar ^1,7^, Nandhakumar Eswaramoorthy^2^, Muthukumarasamy Natarajan^*1^, Agilan Santhanam^1^, Vijayshankar Asokan^3^, Venkatraman Madurai Ramakrishnan^1,7^, Balasundaraprabhu Rangasamy^4^, Senthilarasu Sundaram^5^, Punniamoorthy Ravirajan^6^, Dhayalan Velauthapillai^*7^

^1^Department of Physics, Coimbatore Institute of Technology, Coimbatore, Tamil Nadu, India.

^2^School of Mechanical Engineering, Vellore Institute of Technology, Vellore, Tamil Nadu, India.

^3^Environmental Inorganic Chemistry, Department of Chemistry and Chemical Engineering Chalmers University of Technology, 412 96 Göteborg, Sweden.

^4^Department of Physics, PSG College of Technology, Coimbatore, Tamil Nadu, India.

^5^Environment and Sustainability Institute, University of Exeter, Penryn, Cornwall TR10 9FE United Kingdom.

^6^Department of Physics, University of Jaffna, Jaffna 40000, Sri Lanka

^7^Faculty of Engineering and Science, Western Norway University of Applied Sciences, 5063 Bergen, Norway.

^*^Corresponding Author: Dhayalan.Velauthapillai@hvl.no; vishnukutty2002@yahoo.co.in.

Phone No.: +47 55 58 77 11; +91 0422 2574071

**2.1.3** **FT-IR spectroscopic analysis for plant extracted porous graphitic carbon**

**Figure S1.** FT-IR spectra for the porous graphitic carbon materials EC-GC4, EC-GC8 and EC-GC10 synthesized using different annealing temperatures.

The strong peak appearing around 1059 cm^-1^ for the sample EC-GC4 could be assigned to the =C-O-C vibrations of ether structures ^1, 2^. Further, the peak at 1575 cm^-1^ can be attributed to the aromatic ring skeleton vibration and the asymmetrical stretching vibration of C=O in carboxylate. For the EC-GC8, we could observe a peak associated with shoulders at 1236 cm^-1^ which is related to the symmetrical stretching vibration^3^. Whereas the sample EC-GC10 heated at 1000°C displays feeble peaks at 2858 cm^-1^ and 2933 cm^-1^ and they correspond to C-H bands which is primarily due to the functionalization of oxygen atoms^4^. The appearance of these new predominant functional group peaks for the EC-GC10 sample may be due to the annealing at high temperature.

**2.1.4 Conductivity Measurements**

**Figure S2.** Conductivity measurements for the prepared graphitic carbon samples (EC-GC4, EC-GC8 and EC-GC10) carried out under room temperature using a (ECOPIA, HMS 2000) Hall Effect system. (inset: the hydraulic pelletized EC-GC samples)

**2.4 Optical behavior of the prepared CH_3_NH_3_PbI_3-x_Cl_x_ perovskite**

In recent years, many researchers are keenly working to improve the crystalline nature and the optical absorbance properties of perovskite active layer. Among them, Jiang et. al., have studied the J-V hysteresis behavior of carbon based PSCs and have reported that by adding methylammonium chloride (MACl) additives (0.45 M) into the methylammonium lead iodide (CH_3_NH_3_PbI_3_) perovskite precursor (1 M) enhances the PCE from 8.74 % to 14.27% due to the improved perovskite crystalline nature^5^. Zhou et. al., have demonstrated the incorporation of chlorine ions into CH_3_NH_3_PbI_3_ using low-cost and facile hydrochloric acid vapor annealing (HAVA) post-treatment for 2 minutes. This induces preferably the formation of [PbCl_6_]^4-^ octahedral complex rather than [PbI_6_]^4-^, leading to the significant increase in its PCE from 14.02% to 17.40% ^6^. Mixed halide perovskite (CH_3_NH_3_PbI_3-x_Cl_x_) films were synthesized by exposing lead iodide (PbI_2_) film in CH_3_NH_3_PbCl_3_ vapor, and this resulted in achieving an improved open-circuit voltage. This could be attributed to the change in the energy band gap value by the incorporation of excess chlorine ions from the vapor which has been reported by Sedighi et. al^7^. Yunlong et. al., have also tuned the energy bandgap (E_g_) value of CH_3_NH_3_PbI_3-x_Cl_x_ from 1.54 eV to 1.59 eV by varying the mole fraction of PbCl_2_ concentration^8^. They have also stated that the role of chlorine composition proved to improve the structural, optical and morphological properties of the perovskite materials and this helps in enhancing the device performance.

**
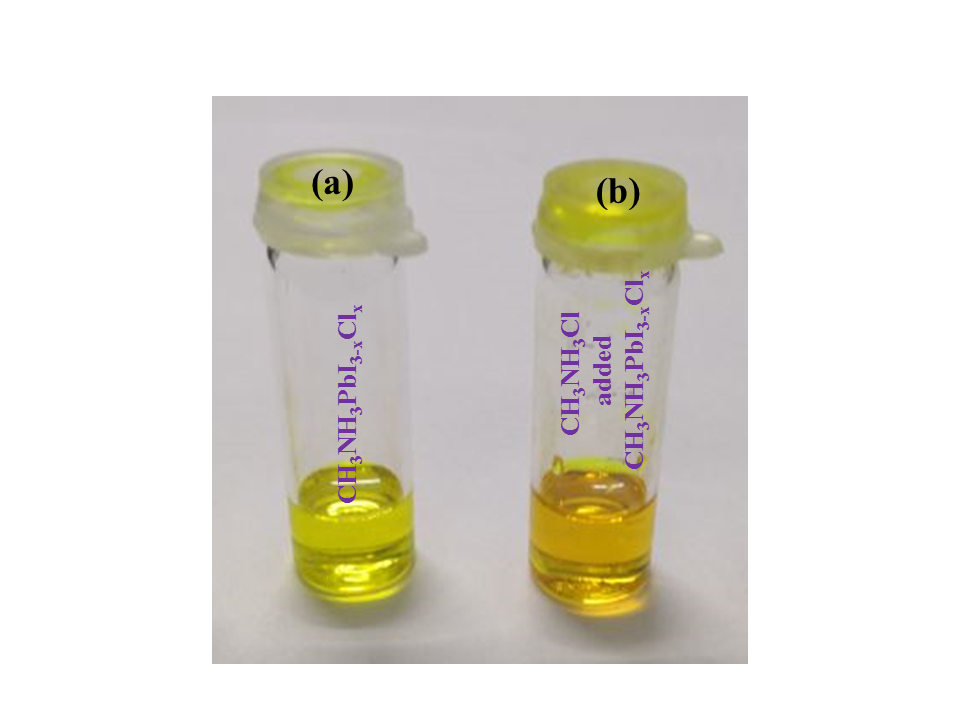
**

**Figure S3.** Digital photograph of (a) the mixed halide perovskite (CH_3_NH_3_PbI_3-x_Cl_x_) solution and (b) excess CH_3_NH_3_Cl added perovskite - CH_3_NH_3_PbI_3-x_Cl_x_ solution.

It is well-known fact from the previous reports that by introducing different organic and inorganic cations, halide anions (I, Cl and Br) and metal cations (Sn, Cu) in perovskite may not only result in improving the PCE but also device stability.^9, 10^ The present study focuses on the compositional engineering to be carried out through the excess addition of halide anion precursor using methylammonium chloride (CH_3_NH_3_Cl) and the optical changes observed has been compared with the prepared CH_3_NH_3_PbI_3-x_Cl_x_ solution. The CH_3_NH_3_Cl added perovskite precursor solution displays a gradual color change from pale yellow to dark yellow (shown in **Figure S3 a & b**) which is attributed to the changes in the absorption wavelength. Consequently, the change in their absorption spectra indicates that there is a change in the energy bandgap (E_g_)^10-14^.

**Figure S4. Optical properties of the prepared perovskite samples:** (a) UV-Vis absorbance spectra of CH_3_NH_3_PbI_3-x_Cl_x_ and CH_3_NH_3_Cl added CH_3_NH_3_PbI_3-x_Cl_x_ perovskite at room temperature (b) Calculated band gap value using Tauc’s plot (c) Emission spectra of the as prepared CH_3_NH_3_PbI_3-x_Cl_x_, CH_3_NH_3_Cl added perovskite - CH_3_NH_3_PbI_3-x_Cl_x_ and porous graphitic carbon encapsulated perovskite (EC-GC10@CH_3_NH_3_PbI_3-x_Cl_x_) using PL studies.

**Figure S4 (a)** illustrates that there is a systematic change in the absorption onset spectra from 826 nm to 843 when the prepared perovskite solution was excessively added with the CH_3_NH_3_Cl solution. The manifested blue shift was identified as due to the signatures of inclusion of extrinsic halide ions from the CH_3_NH_3_Cl into the perovskite lattice^15^ which could be a vital cause in enhancing the stability of the mixed halide perovskite phases.^16-19^ **Figure S4 (b)** shows the observed change in the E_g_ value from ~1.47 eV to ~1.51 eV determined using the Tauc‘s plot relation^20-23^. Similar results were observed by Feng et. al., on the addition of Cl ions into CH_3_NH_3_PbI_3-x_Cl_x_ perovskite for various concentrations of Cl (at x=0, 0.05, 0.33 and 1). From this, a small amount of Cl addition into the perovskite resulted in the broadening of bandgap, an increase in the grain size and improved phase purity. Also, the addition of Cl concentration increases eventually the bandgap value and thereby improves the thermal stability^14^. Thus, the optical properties of the perovskite are greatly influenced by the addition of CH_3_NH_3_Cl and it exhibits a sharply visible spectrum absorption at 826 nm and a suitable bandgap of ~1.51 eV which could be desirable for the use in PSCs and the obtained results are in good agreement with the previous reports^10, 21,24^.

The photoluminescence (PL) emission spectra for the prepared CH_3_NH_3_PbI_3-x_Cl_x_, CH_3_NH_3_Cl added CH_3_NH_3_PbI_3-x_Cl_x_ and EC-GC10@CH_3_NH_3_PbI_3-x_Cl_x_ perovskite is shown in **Figure S4 (c)**. From this, we could infer that the CH_3_NH_3_Cl added perovskite film shows a peak with less PL intensity than the pure perovskite film and the emission onset peak position shows a blue shift that is likely to occur due to the inclusion of localized extrinsic ion defects into the perovskite lattice^25^. The PL spectra of the normal perovskite film shows a sharp emission at 720 nm whereas the luminescence spectra of the EC-GC10@CH_3_NH_3_PbI_3-x_Cl_x_ film has a peak with less intensity and the emission peak is observed at 760 nm^26^ and the reduction in peak intensity may be due to the quenching phenomena originating from the interface of the perovskite and porous graphitic carbon grain boundaries^27^ which may lead to low recombination rate and in turn it might be helpful in improving PSC device performances^28^.

**2.5 Mechanism for the formation of graphitic carbon encapsulated perovskite (EC-GC10@CH_3_NH_3_PbI_3-x_Cl_x_)**

The formation mechanism of EC-GC encapsulated perovskite is shown in **Figure S5 (a)**. From this, it could be observed that the perovskite precursor with the essential chlorine ions generally shows homogenous nucleation ensuring the formation of smooth and fine crystallographic grain textures^24, 29^. Colella et al., have used both the experimental and theoretical calculation to show that incorporation of ‘Cl’ is limited to lower concentrations (below 3-4%) in iodine-based perovskite^30^. Tombe et. al., suggested that the higher halide concentration in the perovskite precursor could affect the device performance ^24^. However, the exact perovskite morphology in terms of the precise effect of chloride concentration is not known as ^31^. Hence, in the present work an addition of 1.5 molar concentration of CH_3_NH_3_Cl solution into the mixed halide perovskite precursor, results in the formation of smooth and uniformly distributed polygon grain structured perovskite films which is expected to provide better light absorption in photovoltaics^9, 32^ and the observed results indicate that it is an ideal active absorber material^10^.

**Figure S5 (b)**, illustrates the mechanism of formation of EC-GC encapsulated perovskite structure (EC-GC10@CH_3_NH_3_PbI_3-x_Cl_x_) on the addition of plant extracted graphitic carbon (EC-GC10) into the homogenous perovskite precursor solution. After the incorporation of EC-GC10 into the perovskite precursor, this EC-GC graphitic carbon acts as heterogeneous nucleation sites for the perovskite crystallization which inhibits the growth of homogenous nucleation sites due to Gibb’s free energy criteria^33^. This phenomenon facilitates the encapsulation of EC-GC over the perovskite materials, in which the residual porous graphitic carbon acts as a capsule that assists in controlling the growth of perovskite nucleation, resulting in effective spatial confinement.

**Figure S5.** Descriptive mechanism on the formation of polygonal structured perovskite-CH_3_NH_3_PbI_3-x_Cl_x_ and encapsulated EC-GC10@CH_3_NH_3_PbI_3-x_Cl_x_.

**2.6 PSC device structure and its work function**

The energy level diagram of the complete carbon-based perovskite device is depicted in **Figure S6 (a)**. Energies are expressed in terms of electron volts (eV), using the electron energy in a vacuum as a reference. The energy levels of the conduction band (CB) edges of FTO, TiO_2_ and perovskite are at -4.0 eV, -4.0 eV and -3.9 eV respectively^34^. Whereas the valence band (VB) edges of TiO_2_ and perovskite are at -7.2 eV and -5.43 eV respectively^35^. The Fermi level of the graphitic carbon is at 5.1 eV ^36^. When the sunlight falls on the working cell, the free electrons are excited up from the CB of the perovskite layer and then jumps to the CB edge of the electron transport TiO_2_ layer and is finally collected at the FTO anode. Meanwhile, the holes formed at the VB of perovskites are extracted by the porous graphitic carbon electrode^37^. Further, we have followed a selective interfacial layer sandwiching between the perovskite/EC-GC carbon which helps in improving electron extraction.

**Figure S6.** Perovskite solar cell (a) Schematic representation of energy level work function diagram of the PSCs (b) cross sectional FE-SEM image of fabricated carbon HTMs based PSCs and (c) Schematic illustration on the encapsulation of EC-GC10@CH_3_NH_3_PbI_3-x_Cl_x_ in the fabricated PSCs.

A typical cross-sectional FE-SEM image of a complete perovskite device architecture is given in **Figure S6 (b)**. The FTO glass is deposited with spray-coated compact and spin-coated porous TiO_2_ blocking layers. Subsequently, the CH_3_NH_3_PbI_3-x_Cl_x_ perovskite thin film layer (~350 nm) is formed on the top of the mesoporous photoanode layer using a one-step solution process. Then, the EC-GC10@CH_3_NH_3_PbI_3-x_Cl_x_ perovskite interfacial layer is coated on the top of the absorber layer using a spin coating method and above this, a hole transport graphitic carbon-based electrode has been coated via brush painting technique. The fabricated structure of the PSC device is glass/FTO/c-TiO_2_/mp-TiO_2_/CH_3_NH_3_PbI_3-x_Cl_x_/EC-GC10@CH_3_NH_3_PbI_3-x_Cl_x_/C-HTMs. **Figure S6 (c)**, shows the schematic illustration of the encapsulated CH_3_NH_3_PbI_3-x_Cl_x_ with EC-GC10. In this, there is strong coordination of Pb^2+^ ion with C=O group of the porous graphitic carbon (due to the availability of 6p electron in the Pb^2+^sites) ^33, 38^ which is believed to be the reason behind the encapsulation mechanism. The incorporation of perovskite into the porous graphitic carbon material paves way for the reduced recombination loss and can thus contribute to increased J_SC_ ^39^.

**2.7 Device stability test for the fabricated PSC devices**

**Figure S7.** Photovoltaic power conversion efficiency of PSC devices fabricated using with and without porous graphitic carbon encapsulated perovskite interfacial layer (EC-GC10@CH_3_NH_3_PbI_3-x_Cl_x_); (a) in terms of efficiency and (b) histogram representation of deterioration value for the device fabricated without interfacial layer (device-1^f^).

**Table S1.** Photovoltaic parameters of PSC devices based on with and without porous graphitic carbon encapsulated perovskite (EC-GC10@CH_3_NH_3_PbI_3-x_Cl_x_) as an interfacial layer measured with an active area of 0.314 sq.cm under 1 sun light (1000mW/cm^2^) illumination.

| **Device Architecture** | **Spin coated (Rpm)** | **Thickness of the Interfacial layer** | **V_OC_ (V)** | **J_SC_ (****mA/cm^2^)** | **FF (%)** | **PCE (%)** |
| --- | --- | --- | --- | --- | --- | --- |
| Device-1^f^ | - | Without interfacial layer | 0.649 | 20.50 | 53.51 | 7.12% |
| Device-1^c^ | 3500 | 80 nm | 0.672 | 23.49 | 54.03 | 8.52% |
| Device-1^g^ | 1500 | 150 nm | 0.719 | 18.81 | 55.05 | 7.44% |

Device- 1^c^ - FTO/c-TiO_2_/mp-TiO_2_/ EC-GC10@CH_3_NH_3_PbI_3-x_Cl_x_ (80 nm)/EC-GC10

Device- 1^f^ - FTO/c-TiO_2_/mp-TiO_2_/ CH_3_NH_3_PbI_3-x_Cl_x_/ porous graphitic carbon

Device- 1^g^ - FTO/c-TiO_2_/mp-TiO_2_/ EC-GC10@CH_3_NH_3_PbI_3-x_Cl_x_ (150 nm)/EC-GC10

**Table S2.** Comparative analysis on surface area, pore size and volume of graphitic carbons extracted from various bio-waste materials

| **Source** | **Material** | **S_BET_^a^**  **(m^2^/g)** | **Pore size (nm)** | **Pore volume (cc/g)** | **Reference** |
| --- | --- | --- | --- | --- | --- |
| Invasive  plant species  (***Eichhornia Crassipes***) | EC-GC10 | 1000.452 | 19.9 | 0.23 | **Present work** |
|  | EC-GC8 | 329.999 | 45.8 | 0.11 |  |
|  | EC-GC4 | 23.941 | 77.3 | 0.29 |  |
| Wood | Graphitic Carbon | 350.95 | - | 0.24 | Wang et. al.,^40^ |
| Cotton |  | 431.29 |  | 0.26 |  |
| Filter paper |  | 293.19 |  | 0.33 |  |
| Sawdust | FeS@GCC composite | 109.3 | - | 0.22 | Haridas et. al.,^41^ |
| Polyacrylic resin | GC@Fe_3_C | 132.44 | - | - | Liao et. al.,^42^ |
| Human Hair | Graphitic Porous Carbon (GPC_850_) | 623 | - | - | Sahasrabudhe et. al., ^43^ |
| Citric acid | Nitrogen-Doped Mesoporous Graphitic Carbon | 204.8 | 3.38 | - | Li et. al., ^44^ |
| Ionic liquids | Nitrogen-Doped Graphitic Carbon | 906 |  |  | Paraknowitsch et. al., ^45^ |

S_BET_ – (Brunauer-Emmett-Teller) surface area.

**Table S3.** Comparative studies on some of the recent reports based on different carbon material as HTMs in perovskite solar cells

| **Device Architecture** | **V_OC_ (V)** | **J_sc_ (mA/cm^2^)** | **FF (%)** | **PCE (%)** | **Reference** |
| --- | --- | --- | --- | --- | --- |
| FTO/c-TiO_2_/mp-TiO_2_/ EC-GC10@CH_3_NH_3_PbI_3-x_Cl_x_/**porous graphitic carbon** | 0.672 | 23.49 | 54.03 | 8.52% | **Present work** |
| FTO/c-TiO_2_/ TiO_2_/MAPbI_3_/ZrO_2_/**carbon** | 0.841 | 15.3 | 65.7 | 8.47% | Hu et. al., ^46^ |
| FTO/TiO_2_/mp-TiO_2_/Perovskite/ Al_2_O_3_/ **CSCNTs**/PMMA | 0.823 | 15.81 | 0.64 | 8.35% | Luo et. al., ^47^ |
| FTO/c-TiO_2_/mp-TiO_2_/Perovskite/**commercial carbon** | 0.90 | 16.78 | 0.55 | 8.31% | Cai et. al., ^48^ |
| Ti foil/TiO_2_ NTs/perovskite/spiro-OMeTAD/**CNTs** | 0.99 | 14.36 | 0.68 | 8.31% | Wang et. al., ^49^ |
| FTO/ZnO dense layer/perovskite/ZnO NR layer/ZrO_2_ layer/ **carbon layer** | 0.960 | 14.82 | 0.58 | 8.23% | Wang et. al., ^50^ |
| FTO/TiO_2_/mp-TiO_2_/CH_3_NH_3_PbBr_3_/**carbon** | 1.35 | 8.35 | 0.72 | 8.09% | Chen et. al., ^51^ |
| FTO/TiO_2_/mp-TiO_2_/porous Al_2_O_3_/Perovskite/**carbon** | 0.78 | 15.1 | 0.68 | 8.0% | Xu et. al., ^52^ |
| FTO/c-TiO_2_/ TiO_2_/MAPbI_3_/**CNT** | 0.703 | 18.54 | 60 | 7.83% | Liu et. al., ^53^ |
| FTO/TiO_2_/mp-TiO_2_/perovskite/**boron and phosphorus co-doped carbon**/Al foil | 0.81 | 18.40 | 0.50 | 7.29% | Yue et. al., ^54^ |

**References**

1. Wang, J. N.; Zhang, L.; Niu, J. J.; Yu, F.; Sheng, Z. M.; Zhao, Y. Z.; Chang, H.; Pak, C., Synthesis of high surface area, water-dispersible graphitic carbon nanocages by an in situ template approach. *Chemistry of materials* **2007,** *19* (3), 453-459.

2. Lu, A.-H.; Li, W.-C.; Salabas, E.-L.; Spliethoff, B.; Schüth, F., Low temperature catalytic pyrolysis for the synthesis of high surface area, nanostructured graphitic carbon. *Chemistry of materials* **2006,** *18* (8), 2086-2094.

3. Miao, M.; Zuo, S.; Zhao, Y.; Wang, Y.; Xia, H.; Tan, C.; Gao, H., Selective oxidation rapidly decomposes biomass-based activated carbons into graphite-like crystallites. *Carbon* **2018,** *140*, 504-507.

4. Choi, D.; Jang, D.; Joh, H.-I.; Reichmanis, E.; Lee, S., High Performance Graphitic Carbon from Waste Polyethylene: Thermal Oxidation as a Stabilization Pathway Revisited. *Chemistry of Materials* **2017,** *29* (21), 9518-9527.

5. Jiang, H.; Liu, X.; Chai, N.; Huang, F.; Peng, Y.; Zhong, J.; Zhang, Q.; Ku, Z.; Cheng, Y.-b., Alleviate the J–V hysteresis of carbon-based perovskite solar cells via introducing additional methylammonium chloride into MAPbI 3 precursor. *RSC Advances* **2018,** *8* (61), 35157-35161.

6. Zhou, W.; Zhou, P.; Lei, X.; Fang, Z.; Zhang, M.; Liu, Q.; Chen, T.; Zeng, H.; Ding, L.; Zhu, J.; Dai, S.; Yang, S., Phase Engineering of Perovskite Materials for High-Efficiency Solar Cells: Rapid Conversion of CH3NH3PbI3 to Phase-Pure CH3NH3PbCl3 via Hydrochloric Acid Vapor Annealing Post-Treatment. *ACS Appl Mater Interfaces* **2018,** *10* (2), 1897-1908.

7. Sedighi, R.; Tajabadi, F.; Shahbazi, S.; Gholipour, S.; Taghavinia, N., Mixed‐Halide CH3NH3PbI3− xXx (X= Cl, Br, I) Perovskites: Vapor‐Assisted Solution Deposition and Application as Solar Cell Absorbers. *ChemPhysChem* **2016,** *17* (15), 2382-2388.

8. Li, Y.; Sun, W.; Yan, W.; Ye, S.; Peng, H.; Liu, Z.; Bian, Z.; Huang, C., High-Performance Planar Solar Cells Based On CH3NH3PbI3-xClx Perovskites with Determined Chlorine Mole Fraction. *Advanced Functional Materials* **2015,** *25* (30), 4867-4873.

9. Bu, X.; Westbrook, R. J. E.; Lanzetta, L.; Ding, D.; Chotchuangchutchaval, T.; Aristidou, N.; Haque, S. A., Surface Passivation of Perovskite Films via Iodide Salt Coatings for Enhanced Stability of Organic Lead Halide Perovskite Solar Cells. *Solar RRL* **2019,** *3* (2), 1800282.

10. Pitchaiya, S.; Natarajan, M.; Santhanam, A.; Asokan, V.; Madurai Ramakrishnan, V.; Selvaraj, Y.; Yuvapragasam, A.; Rangasamy, B.; Sundaram, S.; Velauthapillai, D., The Performance of CH3NH3PbI3 - Nanoparticles based – Perovskite Solar Cells Fabricated by Facile Powder press Technique. *Materials Research Bulletin* **2018,** *108*, 61-72.

11. Pang, S.; Hu, H.; Zhang, J.; Lv, S.; Yu, Y.; Wei, F.; Qin, T.; Xu, H.; Liu, Z.; Cui, G., NH2CH= NH2PbI3: An alternative organolead iodide perovskite sensitizer for mesoscopic solar cells. *Chemistry of Materials* **2014,** *26* (3), 1485-1491.

12. Eperon, G. E.; Stranks, S. D.; Menelaou, C.; Johnston, M. B.; Herz, L. M.; Snaith, H. J., Formamidinium lead trihalide: a broadly tunable perovskite for efficient planar heterojunction solar cells. *Energy & Environmental Science* **2014,** *7* (3), 982-988.

13. Chiang, C.-H.; Nazeeruddin, M. K.; Gratzel, M.; Wu, C.-G., The synergistic effect of H2O and DMF towards stable and 20% efficiency inverted perovskite solar cells. *Energy & Environmental Science* **2017,** *10* (3), 808-817.

14. Xu, F.; Zhang, T.; Li, G.; Zhao, Y., Synergetic Effect of Chloride Doping and CH3NH3PbCl3 on CH3NH3PbI3−xClx Perovskite-Based Solar Cells. *ChemSusChem* **2017,** *10* (11), 2365-2369.

15. Chen, Q.; Zhou, H.; Fang, Y.; Stieg, A. Z.; Song, T. B.; Wang, H. H.; Xu, X.; Liu, Y.; Lu, S.; You, J.; Sun, P.; McKay, J.; Goorsky, M. S.; Yang, Y., The optoelectronic role of chlorine in CH3NH3PbI3(Cl)-based perovskite solar cells. *Nat Commun* **2015,** *6*, 7269.

16. Yang, Y.; Feng, S.; Li, M.; Xu, W.; Yin, G.; Wang, Z.; Sun, B.; Gao, X., Annealing Induced Re-crystallization in CH 3 NH 3 PbI 3− x Cl x for High Performance Perovskite Solar Cells. *Scientific reports* **2017,** *7*, 46724.

17. Xu, F.; Zhang, T.; Li, G.; Zhao, Y., Synergetic Effect of Chloride Doping and CH3NH3PbCl3 on CH3NH3PbI3− xClx Perovskite‐Based Solar Cells. *ChemSusChem* **2017,** *10* (11), 2365-2369.

18. McLeod, J. A.; Wu, Z.; Sun, B.; Liu, L., The influence of the I/Cl ratio on the performance of CH 3 NH 3 PbI 3− x Cl x-based solar cells: why is CH 3 NH 3 I: PbCl 2= 3: 1 the “magic” ratio? *Nanoscale* **2016,** *8* (12), 6361-6368.

19. Tsai, H.; Nie, W.; Cheruku, P.; Mack, N. H.; Xu, P.; Gupta, G.; Mohite, A. D.; Wang, H.-L., Optimizing composition and morphology for large-grain perovskite solar cells via chemical control. *Chemistry of Materials* **2015,** *27* (16), 5570-5576.

20. Pitchaiya, S.; Natarajan, M.; Santhanam, A.; Ramakrishnan, V. M.; Asokan, V.; Palanichamy, P.; Rangasamy, B.; Sundaram, S.; Velauthapillai, D., Nickel sulphide-carbon composite hole transporting material for (CH 3 NH 3 PbI 3 ) planar heterojunction perovskite solar cell. *Materials Letters* **2018,** *221*, 283-288.

21. D'Innocenzo, V.; Srimath Kandada, A. R.; De Bastiani, M.; Gandini, M.; Petrozza, A., Tuning the light emission properties by band gap engineering in hybrid lead halide perovskite. *J Am Chem Soc* **2014,** *136* (51), 17730-3.

22. Ramakrishnan, V. M.; Natarajan, M.; Santhanam, A.; Asokan, V.; Velauthapillai, D., Size controlled synthesis of TiO 2 nanoparticles by modified solvothermal method towards effective photo catalytic and photovoltaic applications. *Materials Research Bulletin* **2018,** *97*, 351-360.

23. Nandhakumar, E.; Priya, P.; Rajeswari, R.; Aravindhan, V.; Sasikumar, A.; Senthilkumar, N., Studies on structural, optical and thermal properties of Fe3O4 (NR)/ZrO2 CSNCs synthesized via green approach for photodegradation of dyes. *Research on Chemical Intermediates* **2019,** *45* (5), 2657-2671.

24. Tombe, S.; Adam, G.; Heilbrunner, H.; Apaydin, D. H.; Ulbricht, C.; Sariciftci, N. S.; Arendse, C. J.; Iwuoha, E.; Scharber, M. C., Optical and electronic properties of mixed halide (X = I, Cl, Br) methylammonium lead perovskite solar cells. *Journal of Materials Chemistry C* **2017,** *5* (7), 1714-1723.

25. Chae, J.; Dong, Q.; Huang, J.; Centrone, A., Chloride Incorporation Process in CH(3)NH(3)PbI(3-x)Cl(x) Perovskites via Nanoscale Bandgap Maps. *Nano letters* **2015,** *15* (12), 8114-21.

26. Ryu, J.; Lee, K.; Yun, J.; Yu, H.; Lee, J.; Jang, J., Paintable Carbon-Based Perovskite Solar Cells with Engineered Perovskite/Carbon Interface Using Carbon Nanotubes Dripping Method. *Small* **2017,** *13* (38).

27. Senthilkumar, N.; Ganapathy, M.; Arulraj, A.; Meena, M.; Vimalan, M.; Vetha Potheher, I., Two step synthesis of ZnO/Ag and ZnO/Au core/shell nanocomposites: Structural, optical and electrical property analysis. *Journal of Alloys and Compounds* **2018,** *750*, 171-181.

28. Tang, H.; Tao, W.; Zhu, B.; Wang, C.; Scarpa, F., Enhanced upconversion luminescence in NaYF 4: Yb, Er nanoparticles by using graphitic carbon shells. *Materials Research Express* **2019**.

29. Yantara, N.; Yanan, F.; Shi, C.; Dewi, H. A.; Boix, P. P.; Mhaisalkar, S. G.; Mathews, N., Unravelling the Effects of Cl Addition in Single Step CH3NH3PbI3 Perovskite Solar Cells. *Chemistry of Materials* **2015,** *27* (7), 2309-2314.

30. Colella, S.; Mosconi, E.; Fedeli, P.; Listorti, A.; Gazza, F.; Orlandi, F.; Ferro, P.; Besagni, T.; Rizzo, A.; Calestani, G.; Gigli, G.; De Angelis, F.; Mosca, R., MAPbI3-xClx Mixed Halide Perovskite for Hybrid Solar Cells: The Role of Chloride as Dopant on the Transport and Structural Properties. *Chemistry of Materials* **2013,** *25* (22), 4613-4618.

31. Edri, E.; Kirmayer, S.; Henning, A.; Mukhopadhyay, S.; Gartsman, K.; Rosenwaks, Y.; Hodes, G.; Cahen, D., Why lead methylammonium tri-iodide perovskite-based solar cells require a mesoporous electron transporting scaffold (but not necessarily a hole conductor). *Nano letters* **2014,** *14* (2), 1000-1004.

32. Dong, H.; Wu, Z.; Xia, B.; Xi, J.; Yuan, F.; Ning, S.; Xiao, L.; Hou, X., Modified deposition process of electron transport layer for efficient inverted planar perovskite solar cells. *Chemical Communications* **2015,** *51* (43), 8986-8989.

33. Zhen, J.; Zhou, W.; Chen, M.; Li, B.; Jia, L.; Wang, M.; Yang, S., Pyridine-functionalized fullerene additive enabling coordination interactions with CH3NH3PbI3 perovskite towards highly efficient bulk heterojunction solar cells. *Journal of Materials Chemistry A* **2019,** *7* (6), 2754-2763.

34. Ku, Z.; Rong, Y.; Xu, M.; Liu, T.; Han, H., Full printable processed mesoscopic CH 3 NH 3 PbI 3/TiO 2 heterojunction solar cells with carbon counter electrode. *Scientific reports* **2013,** *3*, 3132.

35. Zhang, F.; Yang, X.; Wang, H.; Cheng, M.; Zhao, J.; Sun, L., Structure engineering of hole-conductor free perovskite-based solar cells with low-temperature-processed commercial carbon paste as cathode. *ACS Appl Mater Interfaces* **2014,** *6* (18), 16140-6.

36. Mei, A.; Li, X.; Liu, L.; Ku, Z.; Liu, T.; Rong, Y.; Xu, M.; Hu, M.; Chen, J.; Yang, Y., A hole-conductor–free, fully printable mesoscopic perovskite solar cell with high stability. *Science* **2014,** *345* (6194), 295-298.

37. Wei, Z.; Yan, K.; Chen, H.; Yi, Y.; Zhang, T.; Long, X.; Li, J.; Zhang, L.; Wang, J.; Yang, S., Cost-efficient clamping solar cells using candle soot for hole extraction from ambipolar perovskites. *Energy & Environmental Science* **2014,** *7* (10), 3326-3333.

38. Zhang, P.; Kapil, G.; Hamada, K.; Pandey, S. S.; Ma, T.; Hayase, S., Study To Observe the Effect of PbI2 Passivation on Carbon Electrode for Perovskite Solar Cells by Quartz Crystal Microbalance System. *ACS Sustainable Chemistry & Engineering* **2018,** *6* (8), 10221-10228.

39. Matsumoto, F.; Iwai, T.; Moriwaki, K.; Takao, Y.; Ito, T.; Mizuno, T.; Ohno, T., Design of fullerene derivatives for stabilizing LUMO energy using donor groups placed in spatial proximity to the C60 cage. *The Journal of organic chemistry* **2012,** *77* (20), 9038-9043.

40. Wang, C.; Ma, D.; Bao, X., Transformation of biomass into porous graphitic carbon nanostructures by microwave irradiation. *The Journal of Physical Chemistry C* **2008,** *112* (45), 17596-17602.

41. Haridas, A. K.; Jeon, J.; Heo, J.; Liu, Y.; Saroha, R.; Joo, J. H.; Ahn, H.-J.; Cho, K.-K.; Ahn, J.-H., In-situ construction of iron sulfide nanoparticle loaded graphitic carbon capsules from waste biomass for sustainable lithium-ion storage. *ACS Sustainable Chemistry & Engineering* **2019,** *7* (7), 6870-6879.

42. Liao, Y.; Pan, K.; Wang, L.; Pan, Q.; Zhou, W.; Miao, X.; Jiang, B.; Tian, C.; Tian, G.; Wang, G., Facile synthesis of high-crystallinity graphitic carbon/Fe3C nanocomposites as counter electrodes for high-efficiency dye-sensitized solar cells. *ACS applied materials & interfaces* **2013,** *5* (9), 3663-3670.

43. Sahasrabudhe, A.; Kapri, S.; Bhattacharyya, S., Graphitic porous carbon derived from human hair as ‘green’counter electrode in quantum dot sensitized solar cells. *Carbon* **2016,** *107*, 395-404.

44. Li, R.; Cao, A.; Zhang, Y.; Li, G.; Jiang, F.; Li, S.; Chen, D.; Wang, C.; Ge, J.; Shu, C., Formation of nitrogen-doped mesoporous graphitic carbon with the help of melamine. *ACS applied materials & interfaces* **2014,** *6* (23), 20574-20578.

45. Paraknowitsch, J. P.; Zhang, J.; Su, D.; Thomas, A.; Antonietti, M., Ionic liquids as precursors for nitrogen‐doped graphitic carbon. *Advanced Materials* **2010,** *22* (1), 87-92.

46. Hu, R.; Chu, L.; Zhang, J.; Li, X. a.; Huang, W., Carbon materials for enhancing charge transport in the advancements of perovskite solar cells. *Journal of Power Sources* **2017,** *361*, 259-275.

47. Luo, Q.; Ma, H.; Zhang, Y.; Yin, X.; Yao, Z.; Wang, N.; Li, J.; Fan, S.; Jiang, K.; Lin, H., Cross-stacked superaligned carbon nanotube electrodes for efficient hole conductor-free perovskite solar cells. *Journal of Materials Chemistry A* **2016,** *4* (15), 5569-5577.

48. Cai, Y.; Liang, L.; Gao, P., Promise of commercialization: Carbon materials for low-cost perovskite solar cells. *Chinese Physics B* **2018,** *27* (1), 018805.

49. Wang, X.; Li, Z.; Xu, W.; Kulkarni, S. A.; Batabyal, S. K.; Zhang, S.; Cao, A.; Wong, L. H., TiO2 nanotube arrays based flexible perovskite solar cells with transparent carbon nanotube electrode. *Nano Energy* **2015,** *11*, 728-735.

50. Wang, B.; Liu, T.; Zhou, Y.; Chen, X.; Yuan, X.; Yang, Y.; Liu, W.; Wang, J.; Han, H.; Tang, Y., Hole-conductor-free perovskite solar cells with carbon counter electrodes based on ZnO nanorod arrays. *Physical Chemistry Chemical Physics* **2016,** *18* (39), 27078-27082.

51. Chen, H.; Zheng, X.; Li, Q.; Yang, Y.; Xiao, S.; Hu, C.; Bai, Y.; Zhang, T.; Wong, K. S.; Yang, S., An amorphous precursor route to the conformable oriented crystallization of CH 3 NH 3 PbBr 3 in mesoporous scaffolds: toward efficient and thermally stable carbon-based perovskite solar cells. *Journal of Materials Chemistry A* **2016,** *4* (33), 12897-12912.

52. Xu, X.; Zhang, H.; Cao, K.; Cui, J.; Lu, J.; Zeng, X.; Shen, Y.; Wang, M., Lead Methylammonium Triiodide Perovskite‐Based Solar Cells: An Interfacial Charge‐Transfer Investigation. *ChemSusChem* **2014,** *7* (11), 3088-3094.

53. Liu, Z.; Shi, T.; Tang, Z.; Sun, B.; Liao, G., Using a low-temperature carbon electrode for preparing hole-conductor-free perovskite heterojunction solar cells under high relative humidity. *Nanoscale* **2016,** *8* (13), 7017-7023.

54. Yue, G.; Chen, D.; Wang, P.; Zhang, J.; Hu, Z.; Zhu, Y., Low-temperature prepared carbon electrodes for hole-conductor-free mesoscopic perovskite solar cells. *Electrochimica Acta* **2016,** *218*, 84-90.
